# The contribution of depressive ‘disorder characteristics’ to determinations of prognosis for adults with depression: an individual patient data meta-analysis

**DOI:** 10.1017/S0033291721001367

**Published:** 2021-05

**Authors:** Joshua E. J. Buckman, Rob Saunders, Zachary D. Cohen, Phoebe Barnett, Katherine Clarke, Gareth Ambler, Robert J. DeRubeis, Simon Gilbody, Steven D. Hollon, Tony Kendrick, Edward Watkins, Nicola Wiles, David Kessler, David Richards, Deborah Sharp, Sally Brabyn, Elizabeth Littlewood, Chris Salisbury, Ian R. White, Glyn Lewis, Stephen Pilling

**Affiliations:** 1 Centre for Outcomes Research and Effectiveness (CORE), Research Department of Clinical, Educational & Health Psychology, University College London, London WC1E 7HB, UK; 2 iCope – Camden and Islington Psychological Therapies Services, Camden & Islington NHS Foundation Trust, 4 St Pancras Way, London NW1 0PE, UK; 3 Department of Psychiatry, University of California, Los Angeles, Los Angeles, CA 90095, USA; 4 Statistical Science, University College London, London WC1E 7HB, UK; 5 Department of Psychology, School of Arts and Sciences, University of Pennsylvania, Philadelphia, PA 19104-60185, USA; 6 Department of Health Sciences, University of York, York YO10 5DD, UK; 7 Department of Psychology, Vanderbilt University, Nashville, TN 37240, USA; 8 Primary Care, Population Sciences and Medical Education, Faculty of Medicine, University of Southampton, Aldermoor Health Centre, Southampton SO16 5ST, UK; 9 Department of Psychology, University of Exeter, Exeter EX4 4QG, UK; 10 Centre for Academic Mental Health, Population Health Sciences, Bristol Medical School, University of Bristol, Oakfield House, Bristol BS8 2BN, UK; 11 Centre for Academic Primary Care, Population Health Sciences, Bristol Medical School, University of Bristol, Canynge Hall, Bristol, UK; 12 Institute of Health Research, University of Exeter College of Medicine and Health, Exeter EX1 2LU, UK; 13 MRC Clinical Trials Unit, Institute of Clinical Trials and Methodology, University College London, London WC1V 6LJ, UK; 14 Division of Psychiatry, University College London, London W1T 7NF, UK; 15 Camden & Islington NHS Foundation Trust, 4 St Pancras Way, London NW1 0PE, UK

**Keywords:** Depression, individual patient data meta-analysis, prognosis, systematic review, treatment outcome

## Abstract

**Background:**

This study aimed to investigate general factors associated with prognosis regardless of the type of treatment received, for adults with depression in primary care.

**Methods:**

We searched Medline, Embase, PsycINFO and Cochrane Central (inception to 12/01/2020) for RCTs that included the most commonly used comprehensive measure of depressive and anxiety disorder symptoms and diagnoses, in primary care depression RCTs (the Revised Clinical Interview Schedule: CIS-R). Two-stage random-effects meta-analyses were conducted.

**Results:**

Twelve (*n* = 6024) of thirteen eligible studies (*n* = 6175) provided individual patient data. There was a 31% (95%CI: 25 to 37) difference in depressive symptoms at 3–4 months per standard deviation increase in baseline depressive symptoms. Four additional factors: the duration of anxiety; duration of depression; comorbid panic disorder; and a history of antidepressant treatment were also independently associated with poorer prognosis. There was evidence that the difference in prognosis when these factors were combined could be of clinical importance. Adding these variables improved the amount of variance explained in 3–4 month depressive symptoms from 16% using depressive symptom severity alone to 27%. Risk of bias (assessed with QUIPS) was low in all studies and quality (assessed with GRADE) was high. Sensitivity analyses did not alter our conclusions.

**Conclusions:**

When adults seek treatment for depression clinicians should routinely assess for the duration of anxiety, duration of depression, comorbid panic disorder, and a history of antidepressant treatment alongside depressive symptom severity. This could provide clinicians and patients with useful and desired information to elucidate prognosis and aid the clinical management of depression.

## Introduction

Depression is a burdensome disease with a high prevalence, affecting one in 20 adults at any one time (Thornicroft et al., 2017). Not reaching full remission after initial treatment is a strong predictor of poor long-term prognosis including relapse and recurrence of depression (Buckman et al., 2018b; Judd et al., 2000). Knowledge of factors associated with prognosis can be useful for patients and clinicians informing the content of routine clinical assessments and decisions regarding the future clinical management of the patient's condition, and providing them with information they want to know (Trusheim, Berndt, & Douglas, 2007).

There have been a number of different approaches towards studying prognosis. For adults with depression, it has most commonly been studied in systematic reviews or randomised controlled trials (RCTs) that have focused on prognosis for those receiving a single treatment – typically, an antidepressant or cognitive behavioural therapy (Bower et al., 2013*a*, *b*; Chekroud et al., 2016; Driessen, Cuijpers, Hollon, & Dekker, 2010; Karyotaki et al., 2017). Such studies might identify a mixture of general prognostic factors applicable regardless of treatment type and prognostic factors unique to that treatment modality, but due to their design, they cannot distinguish between the two. For example, the predictive models from STAR*D that examined outcomes on the antidepressant citalopram were found to generalise to escitalopram–bupropion but not to venlafaxine–mirtazapine (Chekroud et al., 2016). At the outset of treatment, it is impossible to know what future treatments a patient will receive so general information about prognosis, that would apply to all treatments, is of clinical value (Hippisley-Cox et al., 2007; Trusheim et al., 2007); this can be called ‘prognosis independent of treatment’. Another approach to studying prognostic factors is to identify people with depression from cohort studies. Most cohorts have small numbers of people with depression and many have not sought treatment (Buckman et al., 2018*b*; Hardeveld, Spijker, De Graaf, Nolen, & Beekman, 2009). Therefore, inferences about prognosis from these samples can be imprecise and might not be generalisable to the population of help-seeking patients who are seen by clinicians. The approach taken in the current study is to examine data from the individual participants of a wide range of RCTs that have investigated a breadth of pharmacological, psychological and other interventions, amongst individuals seeking treatment for depression, and to partial out the effects of the randomisation in each study, to investigate the associations between patient characteristics and prognosis. In theory, depending on the breadth of the treatments used in the contributing studies, this approach allows for the investigation of prognostic factors that apply to any course of treatment and should therefore be more generalisable to a wider range of clinical circumstances.

Meta-analyses of individual patient data (IPD) collected from RCTs can provide an improved understanding of factors associated with prognosis independent of treatment (Bower et al., 2013*a*, *b*; Driessen et al., 2010; Gibbons, Hur, Brown, Davis, & Mann, 2012) as they are able to deliver greater power and therefore more precise estimates than individual studies or study-level meta-analyses (Driessen et al., 2010; Fisher, Carpenter, Morris, Freeman, & Tierney, 2017; Stewart et al., 2015). A meta-review of systematic reviews and meta-analyses, including IPD meta-analyses, was conducted to inform the methods and focus of the current study (online Supplementary Tables 1 and 2). That meta-review established that there is strong evidence of an association between the severity of depressive symptoms pre-treatment and prognosis with particular treatments (Bower et al., 2013*a*, *b*; Driessen et al., 2010; Weitz et al., 2015). However, there is uncertainty over the strength and the clinical importance of the association due to a lack of reporting of effect sizes (Chekroud et al., 2016; Noma et al., 2019), and wide confidence intervals (CIs) in the studied effects (Fournier et al., 2010; Johnsen & Friborg, 2015; Weitz et al., 2015). As noted above, there is also the possibility that these associations are limited to patients receiving particular type of treatment only, given the focus of past studies (Bower et al., 2013*a*, *b*; Driessen et al., 2010; Karyotaki et al., 2017). As such, the current evidence may not be useful for clinicians wanting to inform patients of their prognosis before a decision has been made regarding the type of treatment to start, or in settings where the particular treatments studied are not available.

The meta-review identified a number of other potential prognostic factors, including life events, social support and socio-demographics which are beyond the scope of the current study (Buckman et al., 2021), and several others which are related to the severity of the mental health problem a patient with depression might present with in a clinic. These severity-related factors can be referred to as depressive ‘disorder characteristics’, in contrast to depressive ‘symptom severity’. Some of these ‘disorder characteristics’ such as duration and comorbidity with anxiety have been reported to be associated with response to a particular treatment [e.g. citalopram in STAR*D (Chekroud et al., 2016)], but there have been inconsistent findings and most studies did not adjust for depressive symptom severity (Johnsen & Friborg, 2015; Nakabayashi, Hara, & Minami, 2018; Noma et al., 2019). One study has found an interaction between duration and symptom severity suggesting that considerations of prognosis should not be limited to symptom severity alone (Lorenzo-Luaces, Rodriguez-Quintana, & Bailey, 2020). However, that was a study of adolescents, in a single sample, with two treatment types, their combination, or placebo, and was not able to consider a broader spectrum of depressive ‘disorder characteristics’. Previous studies have also rarely included data from primary care settings, or provided insufficient information about how participants were recruited to know if the results are generalisable to other health care settings. Large proportions of adults seeking treatment for depression present in primary care (Olfson, Blanco, & Marcus, 2016; Thornicroft et al., 2017), so identifying prognostic factors in a primary care setting has important utility.

This study aimed to provide clinically useful estimates for prognostic factors that would apply whatever treatment a patient would receive. The specific aims were to investigate: (1) the degree to which depressive symptom severity is associated with prognosis for adults with depression in primary care, independent of treatment type; and (2) which depressive ‘disorder characteristics’ are associated with prognosis independent of treatment type, and independent of depressive symptom severity.

## Methods

This study involved compiling an IPD from RCTs of adults with depression that sought treatment in primary care. In order to thoroughly investigate the association between depressive ‘disorder characteristics’ and prognosis a measure that captures a comprehensive set of such clinical features is required. Scoping searches were conducted to identify the most commonly used measure of this type in RCTs that recruited adults with depression in primary care; we established that this was the Revised Clinical Interview Schedule (CIS-R) (Lewis, Pelosi, Araya, & Dunn, 1992). The CIS-R is a measure commonly used in RCTs and epidemiological studies that has been translated into many languages (McManus, Bebbington, Jenkins, & Brugha, 2016; Subramaniam, Krishnaswamy, Jemain, Hamid, & Patel, 2006). It is used to measure symptoms and make diagnostic determinations of depressive and anxiety disorders in line with criteria from the International Classification of Diseases 10th edition (World Health Organization, 1992). CIS-R is most commonly administered via a computerised program such that lay personnel can conduct the interviews, reducing clinical time and cost (Subramaniam et al., 2006). The use of this measure at baseline was made an inclusion criterion for the searches in order to minimise bias in harmonising data across RCTs. The methods for this systematic review and IPD meta-analysis were pre-registered (Buckman et al., 2020) [PROSPERO: CRD42019129512 (01/04/2019)]; for details of protocol amendments and derivations, see online Supplementary materials.

### Identification and selection of studies

Studies were identified via searches on Medline, Embase, PsycINFO and Cochrane Central (inception to 1st December 2020), hand-searching of reference lists, and contacting experts for unpublished or missed studies. Full details of the searches are provided in online Supplementary Table 3.

#### Inclusion and exclusion criteria

Studies were included if they: were RCTs of adults (aged ⩾16 years) with unipolar depression, or with depressive symptoms significant enough for them to seek treatment, or a CIS-R (Lewis et al., 1992) score of ⩾12 (the usual case definition for a common mental disorder); recruited from primary care; had at least one active treatment arm; and used the CIS-R at baseline.

Studies were excluded if they were studies of: patients with depression secondary to a diagnosis of personality disorder, psychotic conditions or neurological conditions; bipolar or psychotic depressions; children or adolescents; feasibility or were studies of adults with either depression or an anxiety disorder, rather than a primary depression with or without comorbid anxiety.

See Table 1 for details of the included studies.

**Table 1.** Description of studies included in the IPD dataset

Study	*N*	Pragmatic RCT (Y/N)	Inclusion criteria	Age	Gender	T0 Depressive symptom severity	Remission	Interventions	Outcome measure
			Mean (s.d.)	Mean (s.d.)	% Female	Mean (s.d.)	% at 3–4 months		Primary (additional)
AHEAD (Kendrick et al., 2006)	327	Y	Adults with new depressive episodes diagnosed by GP	43.1 (15.4)	67	HADS depression = 10.5 (3.9)	62	TCA *v*. SSRI *v*. lofepramine	HADS (CIS-R)
CADET (Richards et al., 2013)	527	Y	Adults ⩾18, ICD-10 depressive episode	44.4 (13.2)	72	PHQ-9 = 17.7 (5.1)	41	Collaborative care *v*. TAU	PHQ-9
COBALT (Wiles et al., 2013)	469	Y	Adults 18–75 with treatment resistant depression, scoring ⩾14 BDI-II	49.6 (11.7)	72	BDI-II = 31.8 (10.7)	34	CBT + TAU *v*. TAU	BDI-II (PHQ-9)
GENPOD (Wiles et al., 2012)	601	N	Adults 18–74 with depressive episode	38.8 (12.4)	68	BDI-II = 33.7 (9.7)	41	Citalopram *v*. reboxetine	BDI-II (HADS)
HEALTHLINES (Salisbury et al., 2016)	609	Y	Adults ⩾18, PHQ-9 score ⩾10, confirmed diagnosis of depression with CIS-R, internet access	49.5 (12.9)	69	PHQ-9 = 16.9 (4.6)	30	Healthlines telecare + TAU *v*. TAU	PHQ-9
IPCRESS (Kessler et al., 2009)	295	Y	Adults scoring ⩾14 BDI-II and GP confirmed diagnosis of depression	34.9 (11.6)	68	BDI-II = 33.2 (8.8)	34	iCBT + TAU *v*. TAU + waiting list for iCBT	BDI-II
ITAS (Thomas et al., 2004)	798	Y	Adults ⩾16, scored ⩾12 on CIS-R	43.2 (14.8)	68	GHQ = 7.7 (3.2)	N/A; at 6–8 months: 46%	Recommendation + TAU *v*. TAU	GHQ-12
MIR (Kessler et al., 2018)	480	Y	Adults ⩾18 taking SSRIs or SNRIs at adequate dose for ⩾6 weeks, and scored ⩾14 on BDI-II	50.7 (13.2)	69	BDI-II = 31.1 (9.9)	30	Mirtazapine *v*. placebo	BDI-II (PHQ-9)
PANDA (Lewis et al., 2019)	652	Y	Adults presenting with low mood or depression to GP in last 2 years, free of ADM for 8 weeks up to baseline	39.7 (15.0)	59	BDI-II = 23.9 (10.3)	69	Sertraline *v*. placebo	PHQ-9 (BDI-II)
REEACT (Gilbody et al., 2015)	685	Y	Adults with PHQ-9 ⩾10 presenting to GP with depression	39.9 (12.7)	67	PHQ-9 = 16.7 (4.3)	53	Moodgym *v*. beating the blues *v*. TAU	PHQ-9
RESPOND (Sharp et al., 2010)	220	Y	Women meeting criteria for MDD within 6-months post-partum	28.7 (6.4)	100	EPDS = 17.6 (3.4)	56	ADM *v*. listening intervention	EPDS
TREAD (Chalder et al., 2012)	361	Y	Adults 18–69 who met diagnostic criteria for MDD and scored ⩾14 on BDI-II	39.8 (12.6)	66	BDI-II = 32.1 (9.2)	35	Physical activity + TAU *v*. TAU	BDI-II

ADM, antidepressant medication; BDI-II, Beck Depression Inventory; EPDS, Edinburgh Postnatal Depression Scale; GHQ-12, General Health Questionnaire 12 item version; HADS-D, Hospital Anxiety and Depression Scale-depression subscale; iCBT, internet based therapist delivered cognitive behavioural therapy; MDD, major depressive disorder; T0, baseline; TAU, treatment as usual; TCA, tricyclic antidepressant.

### Measures

The measures of depressive symptoms used to determine depressive ‘symptom severity’ and outcomes are noted in Table 1; details of all measures are given in online Supplementary Table 4.

### Ethical considerations and trial registrations

All included studies were granted ethical approvals and all participants gave informed consent (online Supplementary Table 5). No additional NHS ethical approval was required for this study: HRA reference 712/86/32/81.

### Data analysis plan

Details on determining study inclusion, data extraction, data handling and data management, risk of bias and study quality, secondary outcomes and sensitivity analyses, and results from these, are provided in online Supplementary materials.

#### Primary outcomes

The primary outcome was depressive symptoms at 3–4 months. This was captured in two ways: (1) *z*-score (standardised mean) of the scores on the four depressive symptom measures used at 3–4 months post-baseline in each study (Table 1). The score at 3–4 months was divided by the standard deviation for that measure calculated at 3–4 months. (2) The logarithm of depression scale scores irrespective of the measure used. Exponentiation of the regression coefficient provides an estimate of the percentage difference in symptoms.

It was expected that these methods would give broadly similar results but that the log outcome might have greater clinical utility as percentage differences might be more easily understood and do not require division by standard deviation estimates.

#### Prognostic indicators under consideration

(1) Depressive symptom severity at baseline taken as scores on the depressive symptom measures is detailed in Table 1.
(2) Depressive ‘disorder characteristics’:

• the sum of the scores on the CIS-R anxiety subscales, and each individual subscale
• the number of comorbid common mental health disorders (CMDs), and each individual disorder
• the duration of depression
• the duration of anxiety individually and averaged across CIS-R anxiety subscales
• a history of depression
• a history of any previous treatment for depression
• a history of antidepressant treatment
• the degree of functional impairment
• alcohol misuse

#### Primary analyses

Two-stage random effects meta-analyses were conducted for each prognostic factor. This approach removes variance due to the different depressive symptom measures used across the studies, removes potential biases by separating within-study from between-study effects, and allows for more simple formations of forest plots and hence for the assessment of heterogeneity than one-stage approaches (Fisher, 2015; Fisher et al., 2017). It does so by analysing effects within each study first, before aggregating across studies. One-stage approaches have been favoured in other IPD meta-analyses as they allow for more complex modelling (Cuijpers et al., 2014; Weitz, Kleiboer, Van Straten, Hollon, & Cuijpers, 2017). However, as no complex modelling was necessary here, the two-stage approach was most suitable for the aims of the current study (Fisher, 2015).

There were three sets of variables adjusted for in models of each outcome built for each prognostic factor:

(1) The ‘disorder characteristic’ adjusted for age, gender and the specific randomised treatment(s) in each study.
(2) As in (1) with the addition of depressive symptom severity.
(3) As in (2) with the addition of covariates specific to each prognostic indicator.

Covariates were added to the models above if they were: independently associated with the outcome and prognostic indicator; not multi-collinear with prognostic indicators in the model; not systematically missing and if they impacted the effect estimate for the association between prognostic indicator and outcome when included compared to when excluded from the model. Two factors considered *a priori* to be important covariates (age and gender) were controlled for in all models.

Final models were built with the primary outcomes adding each prognostic indicator to the model in order of magnitude of effect from model 3 (one-by-one), and removing those no longer significantly associated with prognosis (at the 5% significance level) after adding subsequent factors. If two items were highly collinear the one contributing least to the model was removed. In the final models, ordinal variables were re-categorised to assess the associations with prognosis in clinically meaningful groups (e.g. duration items were re-categorised into durations at baseline of less than or equal to 1 year, and greater than 1 year). The explanatory utility of the final models was assessed by considering the amount of variance in depressive symptom scale scores at 3–4 months explained by the models when adding each variable one-by-one, using the adjusted *R*^2^ statistic; for details of how this was calculated see online Supplementary materials.

Meta-analyses were conducted using DerSimonian and Laird random effects models with the ‘ipdmetan’ package in Stata (Fisher, 2015). For the *z*-score and log outcomes at 3–4 months and 6–8 months (secondary outcome) linear regression models were fitted. Logistic models were fitted for remission (secondary outcome). The degree of heterogeneity was assessed using prediction intervals and its impact was assessed using the *I*^2^ statistic (Higgins, Thompson, Deeks, & Altman, 2003).

## Results

### Characteristics of the included studies

**Fig. 1.**
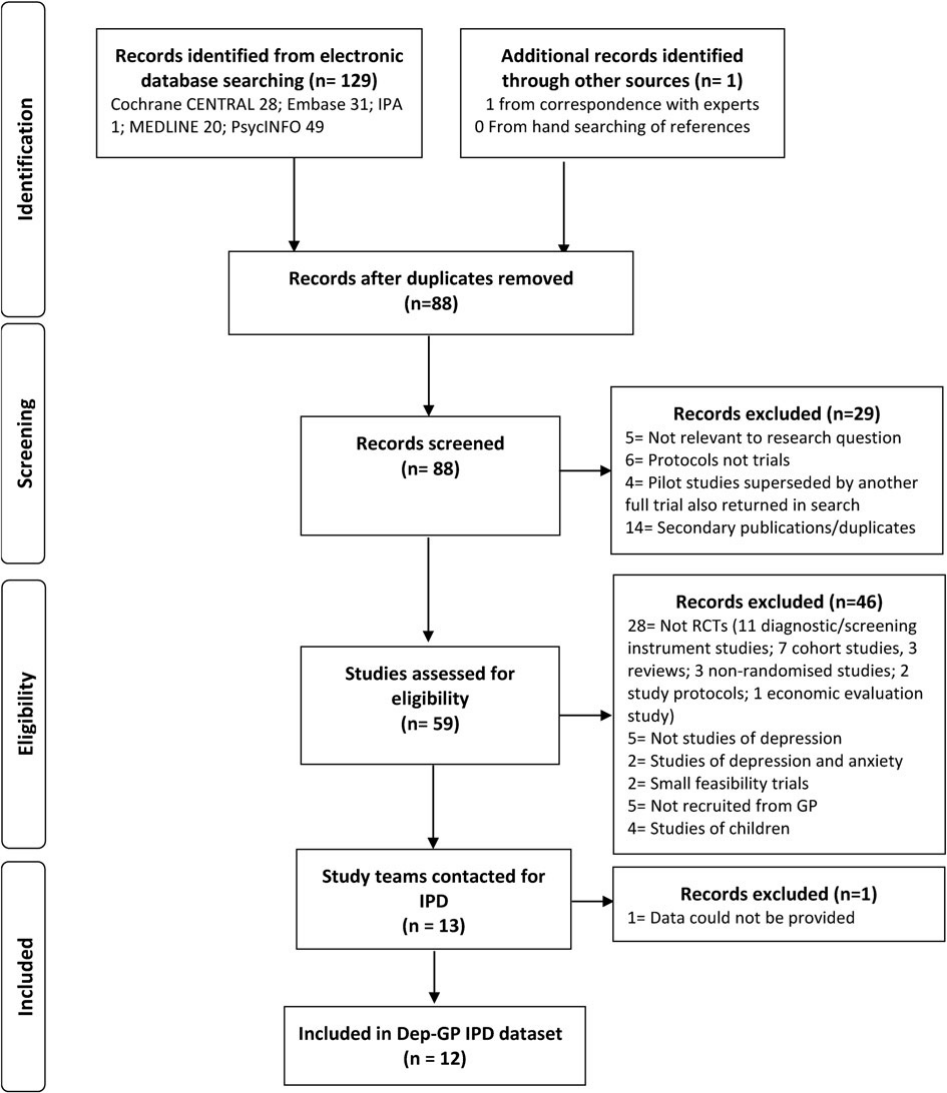
Flowchart of studies through selection process for IPD meta-analysis.

In total, 13 RCTs met inclusion criteria (Fig. 1). Data were not available for one study (Mynors-wallis, Gath, Day, & Baker, 2000). Descriptions of the included studies are given in Table 1. Risk of bias was low in all studies and quality was rated as high (online Supplementary Tables 6 and 7).

A key question in this study was whether or not adjusting for depressive symptom severity ameliorates the associations between depressive ‘disorder characteristics’ and prognosis independent of treatment, therefore descriptive statistics are presented stratified by a median split of depressive symptom severity (Table 2). Means and standard deviations in each strata are presented across all studies. Those with higher depressive symptom severity were more likely to have: identified as female; more comorbid mental health problems; longer durations of their mental health problems; lower social support; lower health-related quality of life; more adverse life events and greater social disadvantages, than those with lower baseline scores (Table 2).

**Table 2.** Baseline characteristics of Dep-GP sample stratified by median split of baseline *z*-score of depressive symptom scale scores using complete data

		Low symptom severity	High symptom severity	χ^2^ or *t* test
Self-reported baseline characteristics	Factor	*N* (%) or mean (s.d.)	*N* (%) or mean (s.d.)	*p* value
**Total**		**2978** (**50)**	**3033** (**50)**	
Age		44.0 (14.6)	41.4 (13.6)	<0.0001
Gender	Female	2005 (67%)	2127 (70%)	0.02
	Male	973 (33%)	906 (30%)	
	Other	0	0	
Ethnicity	White	2262 (94%)	2319 (93%)	0.32
	Non-White	143 (6%)	165 (7%)	
Employment status	Employed	1574 (50%)	1413 (49%)	<0.0001
	Not seeking employment	817 (29%)	749 (26%)	
	Unemployed	385 (14%)	739 (26%)	
Marital status	Married/cohabiting	1333 (55%)	1231 (47%)	<0.0001
	Single	663 (27%)	852 (32%)	
	No longer married	437 (18%)	557 (21%)	
Educational attainment	Degree or higher	691 (32%)	469 (23%)	<0.0001
	A-level or Diplomas	529 (25%)	518 (25%)	
	GCSE	634 (30%)	690 (33%)	
	None or other	289 (14%)	409 (20%)	
Financial status	Doing OK	957 (52%)	581 (32%)	<0.0001
	Just about getting by	573 (31%)	597 (33%)	
	Struggling	322 (17%)	616 (34%)	
Housing status	Home owner	1359 (56%)	1130 (44%)	<0.0001
	Tenant	789 (33%)	1126 (44%)	
	Other	263 (11%)	326 (13%)	
Long-term conditions	No	1653 (75%)	1773 (73%)	0.10
	Yes	539 (25%)	646 (27%)	
Social support	Mean (s.d.)	21.3 (3.3)	19.2 (4.1)	<0.0001
Number of recent life events	Mean (s.d.)	1.4 (1.3)	1.8 (1.5)	<0.0001
AUDIT-PC score	Mean (s.d.)	2.8 (3.0)	2.7 (3.2)	0.71
Hazardous alcohol misuse	No	1224 (79%)	1146 (78%)	
	Yes	327 (21%)	329 (23%)	0.41
EQ5D index score	Mean (s.d.)	0.7 (0.2)	0.7 (0.2)	<0.0001
History of depression	No	815 (29%)	736 (23%)	0.0004
	Yes	1995 (71%)	2415 (77%)	
History of antidepressants	No	1073 (38%)	1035 (33%)	0.0001
	Yes	1739 (62%)	2120 (67%)	
Any past treatment	No	964 (39%)	940 (32%)	<0.0001
	Yes	1519 (61%)	1963 (68%)	
CIS-R total score	Mean (s.d.)	21.9 (8.1)	31.3 (8)	<0.0001
Functional impairment	No impairment	344 (14%)	124 (5%)	<0.0001
	Things more difficult but get everything done	1184 (49%)	902 (34%)	
	Impaired in one activity	376 (15%)	394 (15%)	
	Impaired in more than one activity	533 (22%)	1223 (46%)	
		Mean (s.d.)	Mean (s.d.)	
CIS-R scores	Compulsions	0.6 (1.0)	1.0 (1.3)	<0.0001
	Concentration	1.7 (1.4)	2.4 (1.5)	<0.0001
	Depression	2.3 (1.2)	3.2 (1.0)	<0.0001
	Depressive thoughts	2.5 (1.4)	3.5 (1.1)	<0.0001
	Fatigue	3.0 (1.1)	3.3 (0.9)	<0.0001
	Generalised anxiety	1.8 (1.4)	2.5 (1.5)	<0.0001
	Health anxiety	0.8 (1.1)	1.3 (1.3)	<0.0001
	Irritability	2.0 (1.3)	2.6 (1.3)	<0.0001
	Obsessions	1.0 (1.5)	1.5 (1.7)	<0.0001
	Panic	0.4 (1.0)	1.0 (1.4)	<0.0001
	Phobias	0.9 (1.1)	1.5 (1.4)	<0.0001
	Sleep	1.9 (1.2)	2.6 (1.2)	<0.0001
	Somatic concerns	1.3 (1.4)	1.8 (1.4)	<0.0001
	Worry	2.1 (1.4)	2.8 (1.2)	<0.0001
	Compulsions	1.2 (1.8)	1.5 (1.9)	<0.0001
Duration of symptoms (CIS-R)	Concentration	2.6 (1.6)	3.1 (1.4)	<0.0001
	Depression	3.3 (1.4)	3. 5 (1.3)	<0.0001
	Fatigue	3.1 (1.4)	3.3 (1.3)	<0.0001
	Generalised anxiety	2.3 (1.9)	2.5 (1.9)	0.0001
	Health anxiety	1.8 (1.9)	2.3 (1.9)	<0.0001
	Irritability	2.7 (1.6)	3.0 (1.5)	<0.0001
	Obsessions	1.2 (1.8)	1.7 (1.9)	<0.0001
	Panic	0.6 (1.4)	1.4 (1.9)	<0.0001
	Phobias	1.2 (1.9)	2.0 (2.1)	<0.0001
	Sleep	2.7 (1.8)	3.1 (1.6)	<0.0001
	Somatic concerns	2.3 (1.8)	2.7 (1.7)	<0.0001
	Worry	3.0 (1.6)	3.2 (1.4)	<0.0001
Average anxiety duration		1.8 (0.9)	2.2 (1.0)	<0.0001
Number of comorbid CMHDs		1.5 (1.0)	2.3 (1.1)	<0.0001

*Note*: Numbers do not add up to total *N* due to missing data.

### The association between depressive symptom severity and prognosis independent of treatment

Overall, depressive symptom severity was strongly associated with prognosis at 3–4 months post-baseline. On average, scores at 3–4 months were approximately 31% higher per standard deviation increase in depressive symptoms at baseline (Table 3).

**Table 3.** Outcomes at 3–4 months (‘mean difference’ in *z*-score of depressive symptoms, and percentage difference (%) in depressive symptoms) per unit increase in baseline prognostic indicators

	Adjusted for treatment, age and gender^a^				Additionally adjusted for depressive symptom severity^b^				Additionally adjusted for employment status and/or marital status^c^			
Prognostic indicator	Mean difference (95% CI)	*I*^2^	% (95% CI)	*I*^2^	Mean difference (95% CI)	*I*^2^	% (95% CI)	*I*^2^	Mean difference (95% CI)	*I*^2^	% (95% CI)	*I*^2^
Depressive symptom severity	0.44 (0.41–0.47)	16	30.7 (24.9–36.8)	78	0.44 (0.41–0.47)	16	30.7 (24.9–36.8)	78				
CIS-R total score	0.04 (0.03–0.04)	23	13.1 (10.9–15.4)	62	0.02 (0.01–0.02)	48	6.2 (3.9–8.5)	50				
Depressive subscales total^d^	0.09 (0.08–0.11)	71	27.7 (21.0–34.7)	69	0.04 (0.02–0.05)	40	10.0 (5.0–15.1)	42	0.03 (0.02–0.05)	46	8.2 (3.0–13.7)	45
Anxiety subscales total^e^	0.04 (0.03–0.05)	76	13.5 (10.0–17.1)	71	0.01 (0.01–0.02)	69	4.8 (1.8–8.0)	57	0.01 (0.00–0.02)	68	4.5 (1.4–7.7)	55
Compulsions score	0.13 (0.10–0.17)	44	8.9 (5.8–12.2)	61	0.05 (0.01–0.09)	59	3.6 (0.5–6.8)	63				
Compulsions duration^d^	0.06 (0.04–0.09)	44	4.3 (2.5–6.2)	49	0.03 (0.00–0.05)	43	1.8 (0.1–3.5)	45	0.02 (0.00–0.04)	31	1.7 (0.2–3.3)	35
Concentration score	0.16 (0.11–0.20)	72	9.8 (6.5–13.3)	69	0.05 (0.02–0.07)	0	2.7 (1.0–4.5)	0				
Concentration duration	0.12 (0.09–0.16)	62	8.2 (5.5–10.9)	61	0.06 (0.03–0.09)	49	4.0 (2.0–6.0)	37				
Depression score^f^	0.2 (0.15–0.25)	67	14.0 (10.6–17.5)	55	0.04 (0.01–0.08)	42	3.9 (1.4–6.4)	19	0.03 (−0.01 to 0.07)	42	3.1 (0.2–6.1)	22
Depressive thoughts score^d^	0.22 (0.18–0.25)	52	15.5 (12.2–18.9)	52	0.08 (0.06–0.11)	6	6.3 (3.9–8.8)	19	0.07 (0.04–0.10)	8	4.9 (2.5–7.4)	10
Depression duration^d^	0.15 (0.12–0.18)	27	10.2 (7.4–13.2)	51	0.09 (0.06–0.12)	49	6.6 (4.0–9.3)	49	0.08 (0.05–0.11)	46	5.9 (3.2–8.6)	49
Fatigue score^d^	0.09 (0.03–0.14)	70	6.6 (1.7–11.7)	74	0.03 (0.00–0.07)	30	3.2 (0.2–6.3)	37	0.03 (0.00–0.07)	21	3.2 (0.1–6.3)	31
Fatigue duration	0.12 (0.09–0.15)	35	8.1 (5.3–11.0)	52	0.08 (0.05–0.10)	28	5.7 (3.6–7.9)	25				
Generalised anxiety score^d^	0.10 (0.07–0.13)	33	6.5 (4.4–8.7)	42	0.02 (0.00–0.04)	1	1.3 (−0.3 to 3.0)	7	0.02 (0.00–0.04)	13	1.5 (−0.2 to 3.2)	10
Generalised anxiety duration	0.06 (0.04–0.08)	0	4.4 (2.6–6.3)	32	0.03 (0.01–0.05)	0	2.5 (1.0–4.0)	14				
Health anxiety score^d^	0.15 (0.12–0.18)	41	10.0 (8.0–12.1)	0	0.07 (0.04–0.10)	30	4.3 (2.2–6.5)	18	0.05 (0.02–0.08)	26	3.3 (0.9–5.7)	28
Health anxiety duration^f^	0.08 (0.06–0.09)	1	5.5 (4.2–6.8)	0	0.04 (0.02–0.05)	23	2.8 (1.6–4.0)	1	0.03 (0.01–0.04)	0	2.2 (1.0–3.4)	0
Irritability score^f^	0.09 (0.06–0.11)	34	6.5 (4.5–8.5)	0	0.00 (−0.02 to 0.02)	0	0.7 (−1.3 to 2.7)	10	0.01 (−0.02 to 0.03)	9	1.2 (−0.8 to 3.2)	0
Irritability duration^e^	0.07 (0.04–0.10)	49	5.6 (3.3–8.1)	45	0.03 (0.00–0.06)	53	3.1 (0.8–5.4)	48	0.04 (0.01–0.07)	56	3.3 (0.9–5.7)	50
Obsessions score	0.06 (0.04–0.09)	41	3.5 (1.3–5.6)	52	0.01 (−0.02 to 0.04)	50	0.2 (−1.7 to 2.1)	47				
Obsessions duration	0.05 (0.03–0.07)	0	3.0 (1.4–4.7)	35	0.01 (0.00–0.03)	17	0.7 (−0.8 to 2.2)	34				
Panic score^e^	0.13 (0.07–0.19)	82	8.6 (5.0–12.3)	72	0.05 (0.00–0.09)	72	3.1 (0.3–5.9)	55	0.05 (0.00–0.1)	74	3.1 (0.1–6.2)	59
Panic duration^d^	0.10 (0.08–0.13)	45	6.8 (4.7–8.9)	53	0.05 (0.02–0.07)	36	3.1 (1.4–4.8)	34	0.04 (0.02–0.06)	33	2.6 (1.0–4.3)	35
Phobias score^d^	0.15 (0.10–0.20)	75	10.0 (6.7–13.4)	62	0.06 (0.02–0.11)	64	4.1 (1.4–6.8)	46	0.04 (0.01–0.08)	53	2.9 (0.5–5.4)	32
Phobias duration^f^	0.08 (0.05–0.11)	70	5.6 (3.5–7.7)	63	0.03 (0.01–0.05)	56	2.3 (0.7–3.9)	40	0.02 (0.00–0.05)	52	1.9 (0.4–3.4)	35
Sleep score^f^	0.13 (0.11–0.16)	25	8.3 (6.5–10.1)	45	0.04 (0.01–0.06)	1	2.6 (0.9–4.3)	0	0.02 (0.00–0.05)	10	1.8 (−0.2 to 3.8)	2
Sleep duration^d^	0.10 (0.08–0.13)	35	7.2 (5.1–9.2)	44	0.06 (0.03–0.09)	54	4.6 (2.5–6.7)	51	0.05 (0.03–0.08)	45	4.0 (2.1–6.0)	45
Somatic score^f^	0.10 (0.07–0.13)	50	6.6 (4.1–9.2)	47	0.05 (0.02–0.07)	5	3.2 (1.5–4.8)	0	0.04 (0.02–0.06)	0	2.7 (1.0–4.4)	0
Somatic duration^f^	0.09 (0.07–0.11)	0	6.6 (5.2–8.1)	0	0.05 (0.04–0.07)	0	4.3 (2.9–5.7)	0	0.05 (0.03–0.06)	0	3.8 (2.5–5.2)	0
Worry score^d^	0.11 (0.08–0.14)	36	7.0 (4.9–9.2)	24	0.02 (−0.01 to 0.04)	18	0.9 (−0.8 to 2.7)	0	0.02 (−0.01 to 0.04)	6	0.8 (−1.0 to 2.7)	0
Worry duration	0.09 (0.07–0.11)	0	6.6 (4.6–8.6)	21	0.05 (0.03–0.07)	0	3.7 (2.0–5.4)	0				
Average duration of anxiety^f^	0.26 (0.21–0.31)	58	19.6 (14.6–24.8)	67	0.13 (0.08–0.18)	53	10.3 (6.2–14.5)	51	0.11 (0.07–0.16)	45	9.2 (5.4–13.2)	47
Number of comorbid CMDs^f^	0.21 (0.12–0.29)	87	14.5 (8.5–20.8)	82	0.06 (0.00–0.12)	70	4.6 (0.6–8.7)	56	0.05 (−0.01 to 0.11)	72	4.1 (−0.0 to 8.3)	60
Agoraphobia^d^	0.34 (0.19–0.49)	51	25.7 (16.5–35.7)	11	0.14 (0.02–0.26)	30	10.4 (3.1–18.2)	0	0.09 (−0.03 to 0.20)	25	6.0 (−1.1 to 13.5)	0
CFS^e^	0.31 (0.20–0.43)	64	26.1 (15.6–37.5)	60	0.08 (0.00–0.15)	20	9.8 (4.0–15.9)	0	0.09 (0.01–0.17)	29	10.8 (4.8–17.2)	5
GAD	0.24 (0.18–0.31)	4	17.5 (12.0–23.4)	0	0.04 (−0.02 to 0.10)	0	4.0 (−0.8 to 9.1)	4				
MADD	−0.24 (−0.30 to −0.18)	54	−12.5 (−16.8 to −8.0)	59	−0.05 (−0.12 to 0.01)	0	−2.3 (−7.1 to 2.8)	28				
OCD	0.34 (0.22–0.46)	30	21.5 (13.1–30.6)	9	0.01 (−0.10 to 0.12)	19	−1.3 (−9.2 to 7.3)	29				
Panic disorder	0.41 (0.19–0.64)	72	33.1 (19.1–48.7)	48	0.21 (0.07–0.34)	34	15.0 (6.9–23.7)	0				
Social phobia	0.24 (0.08–0.39)	55	18.3 (6.9–30.8)	47	0.10 (−0.02 to 0.22)	36	8.1 (−0.2 to 17.0)	20				
Specific phobias^d^	0.08 (−0.01 to 0.17)	0	6.3 (−0.0 to 13.0)	0	−0.04 (−0.12 to 0.04)	0	−1.2 (−6.8 to 4.9)	0	−0.02 (−0.10 to 0.06)	0	0.2 (−5.5 to 6.3)	0
History of depression^f^	0.19 (0.12–0.26)	42	8.0 (2.7–13.5)	59	0.11 (0.05–0.17)	30	3.3 (−1.5 to 8.3)	55	0.09 (0.02–0.16)	13	2.6 (−2.6 to 8.2)	53
History of antidepressants	0.17 (0.10–0.25)	20	8.5 (1.9–15.5)	43	0.10 (0.03–0.17)	24	3.7 (−2.1 to 9.9)	40				
Any past treatment	0.19 (0.13–0.26)	0	10.2 (3.9–17.0)	24	0.11 (0.05–0.18)	13	5.2 (−0.7 to 11.4)	26				
Functional impairment^f^	0.27 (0.15–0.39)	72	20.7 (11.3–30.9)	63	0.04 (−0.05 to 0.14)	58	4.6 (−2.4 to 12.0)	48	0.02 (−0.07 to 0.11)	54	2.8 (−3.9 to 9.9)	46
Hazardous alcohol misuse^e^	0.06 (−0.06 to 0.19)	31	7.2 (−4.1 to 19.9)	45	0.01 (−0.09 to 0.10)	0	3.3 (−5.6 to 12.92	28	0.00 (−0.09 to 0.09)	0	2.7 (−5.9 to 12.0)	24

a Adjusted for treatment allocation, age and gender only.

b Adjusted for baseline depression scale *z*-score, age, gender and treatment allocation.

c Additionally adjusted for.

d Employment status.

e Marital status.

f Employment status and marital status.

### Associations between each potential depressive ‘disorder characteristic’ and prognosis

All depressive ‘disorder characteristics’ studied here were associated with prognosis at 3–4 months post-baseline independent of treatment, apart from a comorbid diagnosis of specific phobias, and hazardous alcohol misuse (Table 3). However, after adjustment for baseline depressive symptom severity, there was only evidence of a few ‘disorder characteristics’ being associated with prognosis. Patients with longer durations of depression or of anxiety had poorer prognoses than those with shorter durations. Similarly, patients with a history of depression or treatment for depression had poorer prognoses than those without such histories. However, there was no evidence that functional impairment or most comorbid diagnoses were associated with prognosis after adjusting for depressive symptom severity and covariates (model 3), with the exception of Chronic Fatigue Syndrome, and Panic Disorder.

Findings were consistent when using the *z*-score and log outcomes with one exception in model 3: using the *z*-score there was some evidence that each of the three variables capturing history of depression were associated with prognosis, but no such evidence when using the log outcome.

### Independent associations between depressive ‘disorder characteristics’ and prognosis

Many ‘disorder characteristics’ were missing in two studies (Kendrick et al., 2006; Salisbury et al., 2016). The difference when including or excluding those studies on the effects of variables that were not systematically missing in any study were negligible, see online Supplementary materials. These studies were therefore removed from further primary analyses.

There was only evidence of an association with prognosis for six ‘disorder characteristics’ after adjusting for treatment, depressive symptom severity, covariates and other ‘disorder characteristics’ (online Supplementary Fig. 1). The associations for these six factors were similar across studies with potentially different populations, e.g. in those with ‘treatment resistant depression’ (COBALT), those with apparently less severe depression at baseline (PANDA) and those with postnatal depression (RESPOND); see online Supplementary Fig. 1.

Four ‘disorder characteristics’ were included in the final models in addition to depressive symptom severity (Table 4): duration of depression, average duration of anxiety symptoms, comorbid panic disorder and a history of antidepressant treatment. Although the latter was only significantly associated with prognosis when using the *z*-score outcome, when removing two studies with little variability in this factor due to their inclusion criteria (COBALT and MIR – see Table 1) there was greater evidence for an effect with the log outcome: 6.3% (95% CI: 0.3–12.7). It is noteworthy too that there was 0% heterogeneity in this effect, so there were no substantive differences in the association for studies that randomised to antidepressant treatments and those that did not. The sum of the anxiety subscale scores on CIS-R, and a history of any previous treatment for depression could be included in the final model in place of the average duration of anxiety and a history of antidepressant treatment, respectively, although had weaker associations with outcomes than those retained in the final models (online Supplementary Table 12).

**Table 4.** Association of prognostic indicators with outcomes (mean difference in *z*-score of depressive symptoms and percentage difference (%) in depressive symptoms) after adjusting for disorder characteristics

Prognostic indicator		Independent of treatment and depressive symptom severity^a^				Additionally adjusted for ‘disorder characteristics’^b^			
	High on factor/present *N* (%)	Mean difference (95% CI)^c^	*I*^2^	% difference^d^	*I*^2^	Mean difference (95% CI)^c^	*I*^2^	% difference^d^	*I*^2^
Depressive symptom severity and covariates	2364 (55.10)	0.42 (0.38–0.77)	17	29.9 (22.7–37.6)	78	0.38 (0.34–0.41)	0	26.3 (19.9–33.0)	69
Depression duration^e^	2005 (46.80)	0.18 (0.10–0.23)	29	14.6 (5.9–24.1)	49	0.15 (0.08–0.22)	3	11.5 (3.5–20.1)	36
Average anxiety duration^e^	2780 (64.70)	0.18 (0.09–0.22)	54	14.2 (6.5–22.3)	47	0.12 (0.04–0.21)	37	9.8 (3.6–16.4)	17
Panic disorder	399 (9.30)	0.18 (0.06–0.18)	22	12.5 (4.8–20.8)	0	0.15 (0.03–0.26)	10	9.1 (1.5–17.2)	0
History of antidepressants	2787 (65.00)	0.09 (0.03–0.09)	0	4.5 (−0.7 to 10.0)	0	0.08 (0.01–0.14)	0	3.3 (−1.9 to 8.6)	0

a Adjusted for depressive symptom severity, treatment allocation, age, gender, employment status and marital status.

b Adjusted for depressive symptom severity, depression duration, average anxiety duration, panic disorder, history of antidepressants, treatment allocation, age, gender, employment status and marital status. All models excluded data from AHEAD and HEALTHLINES.

c Using *z*-score at 3–4 months as the outcome.

d Using the natural log of the depressive symptom scale scores at 3–4 months.

e Dichotomised to less than or equal to 1-year, and greater than 1-year duration.

Patients that had durations of depression and anxiety greater than 1 year, had comorbid panic disorder and a history of antidepressant treatment, i.e. those in the ‘high severity’ category on the above variables (*n* = 220), had on average 36.3% (95% CI: 12.4–65.2) higher scores at 3–4 months than patients with none of the above (*n* = 707). Adding all four ‘disorder characteristics’ to models in addition to depressive symptom severity led to substantial gains in the variance explained in the primary outcomes, which increased with each factor added (online Supplementary Table 10).

## Discussion

In this systematic review with IPD meta-analyses it was found that depressive symptom severity was strongly associated with prognosis independent of treatment. Depressive symptom scale scores were on average 31% higher at 3–4 months, 33% higher at 6–8 months and the odds of remission at 3–4 months were approximately halved, for every standard deviation increase in baseline depressive symptoms. Absolute differences were also assessed: for every 11-point increase in BDI-II scores at baseline, scores were about 7 points higher at 3–4 months on average, and for the studies that used the PHQ-9 for each 5-point increase at baseline scores approximately 5 points higher at 3–4 months.

Nearly all ‘disorder characteristics’ were associated with prognosis independent of treatment but only a handful were associated with prognosis independent of depressive symptom severity. This illustrates the importance of adjusting for baseline depression symptom severity when investigating prognosis of depression. The factors independently associated with prognosis were: duration of depression; average duration of anxiety (or severity of anxiety symptoms); comorbid panic disorder and a history of antidepressant treatment (or history of any treatment for depression). The history of treatment variables were not as consistently associated with outcomes as the other factors we identified and the association was relatively small.

There was a lack of evidence for an independent association between functional impairment and prognosis. Functional impairment has been found to be indicative of treatment response for people with either depression or anxiety disorders (Delgadillo, Moreea, & Lutz, 2016; Saunders, Buckman, & Pilling, 2020; Saunders, Cape, Fearon, & Pilling, 2016), so the single item used to capture it here might be insufficient. There was also a lack of evidence to support an association between hazardous alcohol misuse and prognosis, this is in line with previous study that has found it to be related to dropping out of treatment but not to treatment outcomes apart from when patients are alcohol dependent (Boschloo et al., 2012; Buckman et al., 2018*a*, *b*).

### Findings in context

This study provides confirmation that depressive symptom severity is the strongest indicator of prognosis independent of treatment. A number of other studies have found symptom severity to be associated with outcomes but none have considered the association independent of a broad range of commonly available treatments in primary care settings. In addition, given the sample size in this IPD meta-analysis this study was able to address the question of the strength and the clinical importance of the association between symptom severity and prognosis with greater precision than has been possible in other studies (Fournier et al., 2010; Johnsen & Friborg, 2015; Weitz et al., 2015). This study was also the first to comprehensively investigate associations between ‘disorder characteristics’ and prognosis independent of depressive symptom severity. There had been some suggestion from past studies that the duration of depression might be associated with prognosis (Carter et al., 2012; DeRubeis et al., 2014; Fournier et al., 2009; Lorenzo-Luaces et al., 2020; Noma et al., 2019) although there were inconsistencies and contradictory findings in past reviews (see online Supplementary Table 2) (Dodd et al., 2014). In addition, there was limited evidence that comorbid anxiety (Carter et al., 2012; Chekroud et al., 2016) and a history of antidepressant use (Chekroud et al., 2016; Nakabayashi et al., 2018; Saunders et al., 2016) may be associated with outcomes from antidepressant treatments but perhaps not other types of treatment. Here, these were found to be associated with prognosis independent of treatment type, and two novel prognostic factors were also found: the average duration of anxiety problems and comorbid panic disorder.

### Strengths and limitations

There are a number of strengths of the current study. A large dataset was assembled with approximately 98% of the participants in all eligible studies. Over 6000 participants were assessed with the most commonly used comprehensive measure of depressive and anxiety disorders in depression RCTs set in primary care: the (CIS-R), this provided a broad range of prognostic factors to investigate and removed potential biases in harmonising data (Siddique et al., 2015; Weitz et al., 2017). All studies recruited those that had sought treatment in naturalistic, primary care settings. Follow-up rates were generally good and missing data at follow-up had little influence on the findings. A wide range of treatments were used within the randomised studies, including antidepressants, cognitive behavioural therapy of high and low intensities, physical activity and supportive counselling. Causal relationships were not the focus of the current study so confounding was not particularly relevant, but adjustments were able to be made for a number of baseline covariates, adding robustness to the findings. A variety of methods were adopted to assess outcomes and these led to very similar findings, further supporting the conclusions of the current study.

The samples included in this review had been recruited to participate in RCTs, and only studies recruiting in the UK met the inclusion criteria, perhaps due to the use of CIS-R, which may be less familiar to investigators outside the UK. Although it was more commonly used than other clinical interviews, it might have been possible to include studies using those less commonly used interviews too, and then have conducted subgroup analyses per-measure to address issues of harmonising biases. That notwithstanding, there were a number of studies that used the CIS-R and were conducted outside the UK, and a number of studies that used other clinical interviews returned in the scoping searches or full searches for this review, however they often did not meet other inclusion criteria for this review (Husain et al., 2014; Patel et al., 2003). The inclusion of only RCTs and only those that used the CIS-R may have led to a biased sample of all patients with depression and could limit the generalisability of the findings. However, 11 of the 12 studies were pragmatic trials and recruited adults with new episodes of depression, so the participants should be representative of other depressed patients presenting to general practitioners/physicians and psychiatrists across the world. Furthermore, 11 of the 12 studies recruited participants that had actively sought treatment for depression; the other used a variety of methods including recruiting participants as they sought treatment but also calling those that had sought treatment for depression over the previous 2 years pre-baseline and asking if they were willing to be randomised to receive treatment for depression, or a placebo (Lewis et al., 2019). The uniformity of the setting offers an improvement in the extant literature in which there has been limited information about from where participants were recruited (Dal-Ré, Janiaud, & Ioannidis, 2018). Furthermore, there is nothing to suggest that the samples drawn from UK primary care are substantially different to samples of adults with depression seeking treatment elsewhere, and the treatments used in the included studies are common in many countries.

The data on duration were self-reported and relied upon a retrospective judgement; that is likely to have increased measurement error. It is possible that those with more depressive symptoms reported longer durations of illness because of negative cognitive biases, but adjustments were made for baseline depressive symptoms minimising such bias. In any case, knowing that reported duration is a prognostic factor might be of clinical value even if this could be partly influenced by symptom severity (Lorenzo-Luaces et al., 2020).

Heterogeneity in some of the associations was high when considering the *I*^2^ statistic, in the study protocol it was specified that sensitivity analyses would be run where *I*^2^ was above 75% for all factors or above 50% for factors that included in the final models or if there were clear differences between the effects across the studies included in the IPD. More conservative limits for heterogeneity could have been set, but given that none of the sensitivity analyses substantively changed the findings related to any of the prognostic indicators and given that all models were run with random effects for study, it seems unlikely that this would have had a meaningful impact on the results presented here.

### Implications and conclusions

The differences in prognosis observed here were compared with a published estimate for the minimally important clinical difference. Previous study has suggested this is approximately 17.5% in terms of BDI-II scores (Button et al., 2015). The finding that one standard deviation increase in baseline depressive symptoms led to an approximate 31% difference at endpoint therefore suggests that such a change is clinically important. Four additional factors: the duration of anxiety; duration of depression; comorbid panic disorder and a history of antidepressant treatment were also independently associated with poorer prognosis. These depressive ‘disorder characteristics’ are not likely to be associated with clinically important differences when considered alone, but they might be when considered concurrently. For example, those in the ‘high severity’ category of all four factors had outcome symptom scores 36% higher than those in the lowest category. Although this only applied to a small proportion of the patients more than 86% in this sample were in the ‘high severity’ category on at least two of these factors. It may therefore be important for clinicians to assess for all of these factors routinely, pre-treatment. All could be easily captured in clinic or with brief online questionnaires. Assessment of these factors would improve clinicians' ability to predict prognosis.

Future research should ascertain what other factors are informative for prognosis after accounting for depressive ‘disorder characteristics’, whether effects are informative for treatment selection, and whether earlier or more intensive treatments, and more frequent reviews for those likely to have poor outcomes help mitigate these problems, and conversely whether more conservative management is sufficient for those with better prognoses.

## Supplementary material

For supplementary material accompanying this paper visit https://doi.org/10.1017/S0033291721001367. click here to view supplementary material

## References

[ref1] Boschloo, L., Vogelzangs, N., Van Den Brink, W., Smit, J. H., Veltman, D. J., Beekman, A. T. F., & Penninx, B. W. J. H. (2012). Alcohol use disorders and the course of depressive and anxiety disorders. British Journal of Psychiatry, 200(6), 476–484. doi:10.1192/bjp.bp.111.097550. 22322459

[ref2] Bower, P., Kontopantelis, E., Sutton, A., Kendrick, T., Richards, D. A., Gilbody, S., … Liu, E. T.-H. (2013b). Influence of initial severity of depression on effectiveness of low intensity interventions: Meta-analysis of individual patient data. BMJ, 346(feb26 2), f540–f540. doi:10.1136/bmj.f540. 23444423 PMC3582703

[ref3] Bower, P., Kontopantelis, E., Sutton, A., Kendrick, T., Richards, D. A., Gilbody, S., … Liu, E. T. (2013a). Influence of initial severity of depression on effectiveness of low intensity interventions: Meta-analysis of individual patient data. British Medical Journal, 540(February), 1–11. doi:10.1136/bmj.f540. PMC3582703 23444423

[ref4] Buckman, J. E. J., Naismith, I., Saunders, R., Morrison, T., Linke, S., Leibowitz, J., & Pilling, S. (2018a). The impact of alcohol use on drop-out and psychological treatment outcomes in improving access to psychological therapies services: An audit. Behavioural and Cognitive Psychotherapy, 46(5), 513–527. doi:10.1017/S1352465817000819. 29480157 PMC6533638

[ref5] Buckman, J. E. J., Saunders, R., Cohen, Z. D., Clarke, K., Ambler, G., DeRubeis, R. J., … Pilling, S. (2020). What factors indicate prognosis for adults with depression in primary care? A protocol for meta-analyses of individual patient data using the Dep-GP database. Wellcome Open Research, 4, 69. doi:10.12688/wellcomeopenres.15225.3. 31815189 PMC6880263

[ref6] Buckman, J. E. J., Saunders, R., O'Driscoll, C., Cohen, Z. D., Stott, J., Ambler, G., … Pilling, S. (2021). Is social support pre-treatment associated with prognosis for adults with depression in primary care? Acta Psychiatrica Scandinavica, 2021, 1–14. doi:10.1111/acps.13285. PMC7610633 33548056

[ref7] Buckman, J. E. J., Underwood, A., Clarke, K., Saunders, R., Hollon, S. D., Fearon, P., & Pilling, S. (2018b). Risk factors for relapse and recurrence of depression in adults and how they operate: A four-phase systematic review and meta-synthesis. Clinical Psychology Review, 64(7), 13–38. doi:10.1016/j.cpr.2018.07.005. 30075313 PMC6237833

[ref8] Button, K. S., Kounali, D., Thomas, L., Wiles, N. J., Peters, T. J., Welton, N. J., … Lewis, G. (2015). Minimal clinically important difference on the Beck Depression Inventory-II according to the patient's perspective. Psychological Medicine, 45(15), 3269–3279. doi:10.1017/S0033291715001270. 26165748 PMC4611356

[ref9] Carter, G. C., Cantrell, R. A., Zarotsky, V., Haynes, V. S., Phillips, G., Alatorre, C. I., … Marangell, L. B. (2012). Comprehensive review of factors implicated in the heterogeneity of response in depression. Depression and Anxiety, 29(4), 340–354. doi:10.1002/da.21918. 22511365

[ref10] Chalder, M., Wiles, N. J., Campbell, J., Hollinghurst, S. P., Haase, A. M., Taylor, A. H., … Lewis, G. (2012). Facilitated physical activity as a treatment for depressed adults: Randomised controlled trial. BMJ, 344(jun06 1), e2758–e2758. doi:10.1136/bmj.e2758. 22674921 PMC3368484

[ref11] Chekroud, A. M., Zotti, R. J., Shehzad, Z., Gueorguieva, R., Johnson, M. K., Trivedi, M. H., … Corlett, P. R. (2016). Cross-trial prediction of treatment outcome in depression: A machine learning approach. The Lancet Psychiatry, 3(3), 243–250. doi:10.1016/S2215-0366(15)00471-X. 26803397

[ref12] Cuijpers, P., Weitz, E., Twisk, J., Kuehner, C., Cristea, I., David, D., … Siddique, J. (2014). Gender as predictor and moderator of outcome in cognitive behavior therapy and pharamcotherapy for adult depression: An ‘individual patient data’ meta-analysis. Depression and Anxiety, 31, 941–951. doi:10.1002/da.22328. 25407584

[ref13] Dal-Ré, R., Janiaud, P., & Ioannidis, J. P. A. (2018). Real-world evidence: How pragmatic are randomized controlled trials labeled as pragmatic? BMC Medicine, 16(1), 1–6. doi:10.1186/s12916-018-1038-2. PMC5883397 29615035

[ref14] Delgadillo, J., Moreea, O., & Lutz, W. (2016). Different people respond differently to therapy: A demonstration using patient profiling and risk stratification. Behaviour Research and Therapy, 79, 15–22. doi:10.1016/j.brat.2016.02.003. 26937855

[ref15] DeRubeis, R. J., Cohen, Z. D., Forand, N. R., Fournier, J. C., Gelfand, L. A., & Lorenzo-Luaces, L. (2014). The personalized advantage index: Translating research on prediction into individualized treatment recommendations. A demonstration. PLoS One, 9(1), e83875. doi:10.1371/journal.pone.0083875. 24416178 PMC3885521

[ref16] Dodd, S., Berk, M., Kelin, K., Zhang, Q., Eriksson, E., Deberdt, W., & Craig Nelson, J. (2014). Application of the gradient boosted method in randomised clinical trials: Participant variables that contribute to depression treatment efficacy of duloxetine, SSRIs or placebo. Journal of Affective Disorders, 168, 284–293. doi:10.1016/j.jad.2014.05.014. 25080392

[ref17] Driessen, E., Cuijpers, P., Hollon, S. D., & Dekker, J. J. M. (2010). Does pretreatment severity moderate the efficacy of psychological treatment of adult outpatient depression? A meta-analysis. Journal of Consulting and Clinical Psychology, 78(5), 668–680. doi:10.1037/a0020570. 20873902

[ref18] Fisher, D. J. (2015). Two-stage individual participant data meta-analysis and generalized forest plots. The Stata Journal, 2, 369–396.

[ref19] Fisher, D. J., Carpenter, J. R., Morris, T. P., Freeman, S. C., & Tierney, J. F. (2017). Meta-analytical methods to identify who benefits most from treatments: Daft, deluded, or deft approach? Most from particular treatments or other broad approaches used for testing such. BMJ: British Medical Journal, 356(j573), 1–6. doi:10.1136/bmj.j573. PMC5421441 28258124

[ref20] Fournier, J. C., DeRubeis, R. J., Hollon, S. D., Dimidjian, S., Amsterdam, J. D., Shelton, R. C., & Fawcett, J. (2010). Antidepressant drug effects and depression severity. JAMA, 303(1), 47. doi:10.1001/jama.2009.1943. 20051569 PMC3712503

[ref21] Fournier, J. C., DeRubeis, R. J., Shelton, R. C., Hollon, S. D., Amsterdam, J. D., & Gallop, R. (2009). Prediction of response to medication and cognitive therapy in the treatment of moderate to severe depression. Journal of Consulting and Clinical Psychology, 77(4), 775–787. doi:10.1037/a0015401. 19634969 PMC2810269

[ref22] Gibbons, R. D., Hur, K., Brown, C. H., Davis, J. M., & Mann, J. J. (2012). Benefits from antidepressants. Archives of General Psychiatry, 69(6), 572–579. doi:10.1001/archgenpsychiatry.2011.2044. 22393205 PMC3371295

[ref23] Gilbody, S., Littlewood, E., Hewitt, C., Brierley, G., Tharmanathan, P., Araya, R., … White, D. (2015). Computerised cognitive behaviour therapy (cCBT) as treatment for depression in primary care (REEACT trial): Large scale pragmatic randomised controlled trial. British Medical Journal, 351, h5627. doi:10.1136/bmj.h5627. 26559241 PMC4641883

[ref24] Hardeveld, F., Spijker, J., De Graaf, R., Nolen, W. A., & Beekman, A. T. F. (2009). Prevalence and predictors of recurrence of major depressive disorder in the adult population. Acta Psychiatrica Scandinavica, 122, 184–191. Retrieved from http://ovidsp.ovid.com/ovidweb.cgi?T=JS&PAGE=reference&D=psyc6&NEWS=N&AN=2010-15823-002. 20003092 10.1111/j.1600-0447.2009.01519.x

[ref25] Higgins, J. P. T., Thompson, S. G., Deeks, J. J., & Altman, D. G. (2003). Measuring inconsistency in meta-analyses. British Medical Journal, 327, 557–560. 12958120 10.1136/bmj.327.7414.557 PMC192859

[ref26] Hippisley-Cox, J., Coupland, C., Vinogradova, Y., Robson, J., May, M., & Brindle, P. (2007). Derivation and validation of QRISK, a new cardiovascular disease risk score for the United Kingdom: Prospective open cohort study. BMJ, 335(7611), 136. doi:10.1136/bmj.39261.471806.55. 17615182 PMC1925200

[ref27] Husain, N., Chaudhry, N., Fatima, B., Husain, M., Amin, R., Chaudhry, I. B., … Creed, F. (2014). Antidepressant and group psychosocial treatment for depression: A rater blind exploratory RCT from a low income country. Behavioural and Cognitive Psychotherapy, 42(6), 693–705. doi:10.1017/S1352465813000441. 23867053

[ref28] Johnsen, T. J., & Friborg, O. (2015). The effects of cognitive behavioral therapy as an anti-depressive treatment is falling: A meta-analysis. Psychological Bulletin, 141(4), 747–768. doi:10.1037/bul0000015. 25961373

[ref29] Judd, L. L., Paulus, M. J., Schettler, P. J., Akiskal, H. S., Endicott, J., Leon, A. C., … Keller, M. B. (2000). Does incomplete recovery from first lifetime Major depressive episode herald a chronic course of illness? American Journal of Psychiatry, 157, 1501–1504. 10.1176/appi.ajp.157.9.1501 10964869

[ref30] Karyotaki, E., Riper, H., Twisk, J., Hoogendoorn, A., Kleiboer, A., Mira, A., … Cuijpers, P. (2017). Efficacy of self-guided internet-based cognitive behavioral therapy in the treatment of depressive symptoms. JAMA Psychiatry, 74(4), 351. doi:10.1001/jamapsychiatry.2017.0044. 28241179

[ref31] Kendrick, T., Peveler, R., Longworth, L., Baldwin, D., Moore, M., Win, J. C., … Sussex, W. (2006). Cost-effectiveness and cost-utility of tricyclic antidepressants, selective serotonin reuptake inhibitors and lofepramine: Randomised controlled trial. British Journal of Psychiatry, 188(4), 337–345. 10.1192/bjp.188.4.337 16582060

[ref32] Kessler, D., Lewis, G., Kaur, S., Wiles, N., King, M., Weich, S., … Peters, T. J. (2009). Therapist-delivered internet psychotherapy for depression in primary care: A randomised controlled trial. The Lancet, 374(9690), 628–634. doi:10.1016/S0140-6736(09)61257-5. 19700005

[ref33] Kessler, D. S., MacNeill, S. J., Tallon, D., Lewis, G., Peters, T. J., Hollingworth, W., … Wiles, N. J. (2018). Mirtazapine added to SSRIs or SNRIs for treatment resistant depression in primary care: Phase III randomised placebo controlled trial (MIR). BMJ, 363(k4218), k4218. doi:10.1136/bmj.k4218. 30381374 PMC6207929

[ref34] Lewis, G., Duffy, L., Ades, A., Amos, R., Araya, R., Brabyn, S., … Lewis, G. (2019). The clinical effectiveness of sertraline in primary care and the role of depression severity and duration (PANDA): A pragmatic, double-blind, placebo-controlled randomised trial. The Lancet Psychiatry, 6(11), 903–914. doi:10.1016/S2215-0366(19)30366-9. 31543474 PMC7029306

[ref35] Lewis, G., Pelosi, A. J., Araya, R., & Dunn, G. (1992). Measuring psychiatric disorder in the community: A standardized assessment for use by lay interviewers. Psychological Medicine, 22, 465–486. 1615114 10.1017/s0033291700030415

[ref36] Lorenzo-Luaces, L., Rodriguez-Quintana, N., & Bailey, A. J. (2020). Double trouble: Do depression severity and duration interact to predicting treatment outcomes in adolescent depression? Behaviour Research and Therapy, 131, 103637. doi:10.1016/j.brat.2020.103637. 32413595 PMC7984583

[ref37] McManus, S., Bebbington, P., Jenkins, R., & Brugha, T. S. (2016). Mental health and wellbeing in England: Adult psychiatric morbidity survey 2014. Leeds: NHS Digital.

[ref38] Mynors-wallis, L. M., Gath, D. H., Day, A., & Baker, F. (2000). Randomised controlled trial of problem solving treatment, antidepressant medication, and combined treatment for major depression in primary care. British Journal of Psychiatry, 320, 26–30. 10.1136/bmj.320.7226.26 PMC27250 10617523

[ref39] Nakabayashi, T., Hara, A., & Minami, H. (2018). Impact of demographic factors on the antidepressant effect: A patient-level data analysis from depression trials submitted to the pharmaceuticals and medical devices agency in Japan. Journal of Psychiatric Research, 98, 116–123. doi:10.1016/j.jpsychires.2017.12.019. 29334636

[ref40] Noma, H., Furukawa, T. A., Maruo, K., Imai, H., Shinohara, K., Tanaka, S., … Cipriani, A. (2019). Exploratory analyses of effect modifiers in the antidepressant treatment of major depression: Individual-participant data meta-analysis of 2803 participants in seven placebo-controlled randomized trials. Journal of Affective Disorders, 250(December 2018), 419–424. doi:10.1016/j.jad.2019.03.031. 30878654

[ref41] Olfson, M., Blanco, C., & Marcus, S. C. (2016). Treatment of adult depression in the United States. JAMA Internal Medicine, 176(10), 1482–1491. doi:10.1001/jamainternmed.2016.5057. 27571438

[ref42] Patel, V., Chisholm, D., Rabe-hesketh, S., Dias-saxena, F., Andrew, G., & Mann, A. (2003). Efficacy and cost-effectiveness of drug and psychological treatments for common mental disorders in general health care in Goa, India: A randomised, controlled trial. Lancet, 361, 33–39. 12517464 10.1016/S0140-6736(03)12119-8

[ref43] Richards, D. A., Hill, J. J., Gask, L., Lovell, K., Chew-Graham, C., Bower, P., … Barkham, M. (2013). Clinical effectiveness of collaborative care for depression in UK primary care (CADET): Cluster randomised controlled trial. BMJ, 347(aug19 1), f4913–f4913. doi:10.1136/bmj.f4913. 23959152 PMC3746956

[ref44] Salisbury, C., Cathain, A. O., Edwards, L., Thomas, C., Gaunt, D., Hollinghurst, S., … Montgomery, A. A. (2016). Effectiveness of an integrated telehealth service for patients with depression: A pragmatic randomised controlled trial of a complex intervention. The Lancet Psychiatry, 3(6), 515–525. doi:10.1016/S2215-0366(16)00083-3. 27132075

[ref45] Saunders, R., Buckman, J. E. J., & Pilling, S. (2020). Latent variable mixture modelling and individual treatment prediction. Behaviour Research and Therapy, 124(October 2019), 103505. doi:10.1016/j.brat.2019.103505. 31841709 PMC7417810

[ref46] Saunders, R., Cape, J., Fearon, P., & Pilling, S. (2016). Predicting treatment outcome in psychological treatment services by identifying latent profiles of patients. Journal of Affective Disorders, 197, 107–115. doi:10.1016/j.jad.2016.03.011. 26991365

[ref47] Sharp, D. J., Chew-graham, C. A., Tylee, A., Lewis, G., Howard, L., Anderson, I., … Peters, T. J. (2010). A pragmatic randomised controlled trial to compare antidepressants with a community-based psychosocial intervention for the treatment of women with postnatal depression: The RESPOND trial. Health Technology Assessment, 14(43), 1–198. 10.3310/hta14430 20860888

[ref48] Siddique, J., Reiter, J. P., Brincks, A., Gibbons, R. D., Crespi, C. M., & Brown, C. H. (2015). Multiple imputation for harmonizing longitudinal non-commensurate measures in individual participant data meta-analysis. Statistics in Medicine, 34(20), 3399–3414. doi:10.1002/sim.6562.Multiple. 26095855 PMC4596762

[ref49] Stewart, L. A., Clarke, M., Rovers, M., Riley, R. D., Simmonds, M., Stewart, G., … PRISMA-IPD Development Group (2015). Preferred reporting items for a systematic review and meta-analysis of individual participant data. JAMA, 313(16), 1657. doi: 10.1001/jama.2015.3656. 25919529

[ref50] Subramaniam, K., Krishnaswamy, S., Jemain, A. A., Hamid, A., & Patel, V. (2006). The clinical interview schedule-revised (CIS-R)-Malay version, clinical validation. Malaysian Journal of Medical Sciences, 13(1), 58–62. PMC3347904 22589592

[ref51] Thomas, H. V., Lewis, G., Watson, M., Thomas, H. V., Lewis, G., Watson, M., … Sharp, D. (2004). Computerised patient-specific guidelines for management of common mental disorders in primary care: A randomised controlled trial. British Journal of General Practice, 54(508), 832–837. PMC1324916 15527609

[ref52] Thornicroft, G., Chatterji, S., Evans-Lacko, S., Gruber, M., Sampson, N., Aguilar-Gaxiola, S., … Kessler, R. C. (2017). Undertreatment of people with major depressive disorder in 21 countries. British Journal of Psychiatry, 210(2), 119–124. doi:10.1192/bjp.bp.116.188078. PMC5288082 27908899

[ref53] Trusheim, M. R., Berndt, E. R., & Douglas, F. L. (2007). Stratified medicine: Strategic and economic implications of combining drugs and clinical biomarkers. Nature Reviews Neuroscience, 6(April), 287–293. 10.1038/nrd2251 17380152

[ref54] Weitz, E. S., Hollon, S. D., Twisk, J., Van Straten, A., Huibers, M. J. H., David, D., … Cuijpers, P. (2015). Baseline depression severity as moderator of depression outcomes between cognitive behavioral therapy vs pharmacotherapy: An individual patient data meta-analysis. JAMA Psychiatry, 72(11), 1102–1109. doi:10.1001/jamapsychiatry.2015.1516. 26397232

[ref55] Weitz, E., Kleiboer, A., Van Straten, A., Hollon, S. D., & Cuijpers, P. (2017). Individual patient data meta-analysis of combined treatments versus psychotherapy (with or without pill placebo), pharmacotherapy or pill placebo for adult depression: A protocol. BMJ Open, 7(2), 1–8. doi:10.1136/bmjopen-2016-013478. PMC5318563 28193851

[ref56] Wiles, N. J., Mulligan, J., Peters, T. J., Cowen, P. J., Mason, V., Nutt, D., … Lewis, G. (2012). Severity of depression and response to antidepressants: GENPOD randomised controlled trial. British Journal of Psychiatry, 200(2), 130–136. doi:10.1192/bjp.bp.110.091223. 22194183

[ref57] Wiles, N., Thomas, L., Abel, A., Ridgway, N., Turner, N., Campbell, J., … Lewis, G. (2013). Cognitive behavioural therapy as an adjunct to pharmacotherapy for primary care based patients with treatment resistant depression: Results of the CoBalT randomised controlled trial. The Lancet, 381(9864), 375–384. doi:10.1016/S0140-6736(12)61552-9. 23219570

[ref58] World Health Organization (1992). The ICD-10 classification of mental and behavioural disorders: Diagnostic criteria for research. Geneva: World Health Organization.

