# Supplementary material for: The contribution of depressive ‘disorder characteristics’ to determinations of prognosis for adults with depression: an individual patient data meta-analysis
Source: Psychol Med. 2021 Apr 14;51(7):1068–81. doi: 10.1017/S0033291721001367 (PMC8188529; doi:10.1017/S0033291721001367)
Supplement: Supplementary file 1 [file S0033291721001367sup001.docx]

**Supplementary Materials**

Contents

[Details of review of systematic reviews and meta-analyses 2](#_Toc51058528)

[Details of search terms and search results for RCTs to form IPD dataset 14](#_Toc51058529)

[Identification and selection of studies 15](#_Toc51058530)

[Risk of Bias 15](#_Toc51058531)

[Patient and Public Involvement 15](#_Toc51058532)

[Details of additions, deviations and changes to protocols 15](#_Toc51058533)

[Secondary Outcomes 16](#_Toc51058534)

[Sensitivity Analyses 16](#_Toc51058535)

[Calculating variance explained in the primary outcome 16](#_Toc51058536)

[Ethical Approvals and Trial Registrations details for studies included in Dep-GP IPD dataset 18](#_Toc51058537)

[Data Integrity Checks 19](#_Toc51058538)

[Quality assessments and Risk of Bias 19](#_Toc51058539)

[Additional Details of Data Analyses 21](#_Toc51058540)

[Additional Results of Sensitivity Analyses 21](#_Toc51058541)

[Additional References 34](#_Toc51058542)

## Details of review of systematic reviews and meta-analyses

Searches were run on the Cochrane database of systematic reviews, the Prospero register of systematic reviews, Embase, and Medline. Details of the search terms and results from the searches can be found in Supplementary Table 1. Across the databases 632 articles remained after removing duplicates, 71 of these were somewhat relevant to the aim of this thesis and were read in full, from which 29 were directly relevant as they identified patient characteristics associated with prognosis for adults with depression. These 29 studies are summarised in Supplementary Table 2.

As can be seen in Supplementary Table 2 none of the studies investigated factors associated with prognosis independent of a range of treatments (i.e. after adjusting for treatment type in models of outcome), 22 assessed response to particular treatments, six were studies of the “natural course” of depression (i.e. prognosis in untreated samples, although three of these included analyses irrespective of treatment (i.e. ignoring treatment type in those that received some treatment), whether intentionally or not), and one aimed to assess prognosis irrespective of treatment.

**Supplementary Table 1.** Bibliographic database searches and results for literature review on indicators of prognosis for adults with depression

| **Searches** | **Results** |
| --- | --- |
| **Cochrane Database of Systematic Reviews (last searched on 27^th^ February 2020)** |  |
| 1. (depression or MDD or Major Depression or depressive episode): ti.ab.kw (Word variations have been searched) | 656 |
| 2. AND (Prognosis or Outcome): ti.ab.kw (Word variations have been searched) | 574 |
| 3. AND (systematic review or meta-analysis or meta analysis): ti,ab,kw (Word variations have been searched) | 350 |
| 4. NOT (psychosis or bipolar or bi-polar) | 330 |
| 5. Limit to Topic “Mental Health” | **131** |
| **Prospero (last searched on 27^th^ February 2020)** |  |
| 1. (depression or Depressive disorder or Major depression or Unipolar depression or MDD) | 5785 |
| 2. Filter in Health area of review “Mental health and behavioural conditions, or Public health (including social determinants of health) | 2302 |
| 3. Filter in Type and method of review “Epidemiologic, Prognostic, Systematic Review, Meta-analysis, Individual patient data (IPD) Meta-analysis, Network meta-analysis, Review of reviews, or Qualitative synthesis” | 2254 |
| 4. Filter in Status of Review “Published” | **136** |
| **Embase searched 1974 to 2020 February 28** |  |
| 1. (Major depression or MDD or Major Depressive Disorder).m_titl. | 18745 |
| 1. (minor depression or MinD).m_titl | 13201 |
| 1. (depressive or depressive episode or depressive disorder).m.titl | 39836 |
| 1. Depression.m_titl. | 125022 |
| 1. 1 or 2 or 3 or 4 | 176324 |
| 1. treatment outcome/ | 839098 |
| 1. treatment response.mp. or treatment response/ | 271731 |
| 1. prognosis.mp. or prognostic.m[p. or prognostic assessment/ | 4939326 |
| 1. moderator | 8831 |
| 1. systematic review.mp. or “systematic review”/ or meta analysis/ | 385120 |
| 1. 2 or 3 or 4 or 5 | 1477081 |
| 1. 1 and 6 and 7 | 1251 |
| 1. (children or adolescent or child).mp. [mp=title, abstract, heading word, drug trade name, original title, device manufacturer, drug manufacturer, device trade name, keyword, floating subheading word, candidate term word] | 3167378 |
| 1. 12 NOT 13 | 1149 |
| 1. (old age or geriatric).mp. [mp=title, abstract, heading word, drug trade name, original title, device manufacturer, drug manufacturer, device trade name, keyword, floating subheading word, candidate term word] | 142854 |
| 1. 14 NOT 15 | 1106 |
| 1. bipolar disorder/ or bipolar depression/ or bipolar.mp. or psychosis.mp. or psychotic.mp. or schizoaffective.mp. or schizophrenia.mp. [mp=title, abstract, heading word, drug trade name, original title, device manufacturer, drug manufacturer, device trade name, keyword, floating subheading word, candidate term word | 355747 |
| 1. 16 NOT 17 | 962 |
| 1. (stroke or dementia or parkinson* disease or brain injury).mp. [mp=title, abstract, heading word, drug trade name, original title, device manufacturer, drug manufacturer, device trade name, keyword, floating subheading word, candidate term word] | 893303 |
| 1. 18 NOT 19 | 900 |
| 1. Limit 20 to (human and English and journal) | **864** |
| **Ovid MEDLINE 1946 to March Week 3 2019 (last searched on 27^th^ February 2020)** |  |
| 1. exp major depression/ or exp "depression (emotion)"/ or exp Depressive Disorder/ | 115136 |
| 2. prognosis.mp. | 726849 |
| 3. exp Treatment outcome/ | 1028415 |
| 4. 2 or 3 | 1656920 |
| 5. systematic review.mp. or “Systematic Review”/ | 169051 |
| 6. meta-analysis.mp. or Meta-Analysis/ | 179131 |
| 7. 5 or 6 | 271962 |
| 8. 1 and 4 and 7 | 578 |
| 9. limit 8 to (English language and humans and "reviews (maximizes specificity)") | **513** |

**Supplementary Table 2.** Review of systematic reviews, meta-analyses and IPD studies that report on the associations between patient characteristics and prognosis.

| **Article** | **Study Type** | **Studies searched for** | **Number of studies of depression (K for meta) and sample size (N for meta)*** | **Sample, Setting, Recruitment** | **Type of Prognosis Studied** | **Baseline Patient Prognostic Factors Assessed** | **Main findings regarding prognostic factors** | **Limitations for assessing factors associated with prognosis** |
| --- | --- | --- | --- | --- | --- | --- | --- | --- |
| Noma et al., 2019 (Noma et al., 2019) | Not SR but used IPD | Placebo-controlled RCTs conducted in Japan | K=7(7)  N=2803(2803) | Unclear | Response to a particular treatment (antidepressants), prescriptive test of interaction | Depressive symptom severity, duration of episode, number of past episodes, age, gender, and age at onset | Depressive symptom severity was associated with outcome both in antidepressant and placebo groups, duration was only associated with outcome in the antidepressant group, age, age at onset, and gender were only associated with outcome in the placebo group. No association between having 3 or more past episodes and prognosis in either treatment condition. Reviewers rated risk of attrition bias as low in 5 studies and unclear in 2 studies. | Different outcome measures were converted via a crosswalk. Two-stage meta-analysis adjusted for treatment but no assessment of heterogeneity making results difficult to interpret. Did not adjust for baseline depressive symptom severity in analyses of other prognostic variables. |
| Marwood et al., 2018 (Marwood, Wise, Perkins, & Cleare, 2018) | Study-level meta-analysis | Neuroimaging studies of patients with depression or anxiety | K=4(2)  N=86(33) | Unclear setting; Sample: adults with depression or anxiety scanned before starting treatment | Prognosis irrespective of treatment | Connectivity in neural regions | Too few studies to assess disorder specific effects or make appropriate comparisons between them. Greater activation of the right cuneus cortex at baseline was associated with greater symptomatic improvement across disorders | Very limited by small sample size and small number of studies, particularly of depression. Heterogeneity could not be interpreted. No consideration of attrition. No adjustment for baseline depressive symptom severity. |
| Wang et al., 2018 (Wang, Mann, Lloyd-Evans, Ma, & Johnson, 2018) | Systematic Review | longitudinal studies | K=23(N/A)  N= N/S | Unclear | Natural course | Loneliness, social support | 20 studies of depression assessed depressive symptoms at endpoint: lower social support at baseline was associated with higher depressive symptoms at end-point, likewise with higher self-rated loneliness and outcome (in one study) | No quantitative synthesis, uncertainty of sample size, setting and measures used to determine outcomes in the studies qualitatively synthesised. Findings are therefore hard to interpret and associations were most often not adjusted for any treatment. Only 9 studies adjusted for baseline depressive symptoms though findings were still significant in those studies. Attrition rate was unclear in 5 studies and was above 20% in 11 studies. |
| Haq et al., 2015 (Haq, Sitzmann, Goldman, Maixner, & Mickey, 2015) | Study-level meta-analysis | Studies of ECT published since 1980 | K=51(7 to 32)  N= N/S (702 to 1175) | Unclear | Response to a particular treatment (ECT) | Depressive symptom severity, duration of depression, history of treatment with antidepressants, age at onset, age, gender | Shorter durations of depression (7 studies, n=702) were associated with better response to ECT, history of failure to respond to antidepressants (11 studies, n=1175) was associated with worse prognosis. Age was weakly associated with response but there was considerable heterogeneity. Bipolar diagnosis, sex, age at onset, and number of previous episodes were not significant predictors. Association of depressive symptom severity and response to ECT was inconclusive due to high heterogeneity. | Some analyses limited to small number of studies, no weighting for different types of study or sensitivity analyses excluding studies with patients with bi-polar or psychotic depressions making heterogeneity hard to interpret. No assessment of study attrition. Did not adjust for baseline depressive symptom severity in analyses of other prognostic variables. |
| Johnsen & Friborg, 2015 (Johnsen & Friborg, 2015) | Study-level meta-analysis | Empirical studies of CBT including RCTs, non-randomised studies, uncontrolled studies, and clinical field studies. | K=70(70)  N=2426(2426) | Unclear | Response to a particular treatment (CBT), interactions with time/publication year were also tested | Depressive symptom severity, age and gender | Age was not related to variation in treatment effects but gender was (studies of women only had higher effect sizes). The number of comorbid diagnoses was not related to outcomes. The severity of the depressive diagnosis was not associated with outcomes but lower effect sizes were found in studies of mild depression. There were no significant interactions between patient-level characteristics and publication year. | No differential weighting or sensitivity analyses treating the different types of studies differently. Combination of BDI-I and BDI-II scores may have introduced bias particularly to assessment of temporal effects. Recovery was also defined differently across studies making heterogeneity hard to interpret. No assessment of study attrition. Did not adjust for baseline depressive symptom severity in analyses of other prognostic variables. |
| Sugarman et al., 2014 (Sugarman, Loree, Baltes, Grekin, & Kirsch, 2014) | Study-level meta-analysis | Industry sponsored placebo-controlled RCTs of Paroxetine registered with FDA for Depression or Anxiety | K=27(27)  N=4986(4986) | Unclear | Response to a particular treatment (antidepressants), prescriptive test of interaction | Depressive symptom severity | Higher baseline symptom severity was associated with smaller pre-post effect sizes in both Paroxetine and placebo groups, there was no significant treatment type by severity interaction though. | Included one study of adolescents and two of geriatric populations. Average depression severity ranged from severe to very severe in all studies. Heterogeneity relatively high in both drug and placebo groups. No assessment of study attrition. |
| Dodd et al., 2014 (Dodd et al., 2014) | IPD | Industry sponsored placebo-controlled RCTs of Duloxetine | K=12(12)  N=4987(4987) | Unclear | Response to a particular treatment (antidepressants) | Depressive symptom severity, duration of episode, number of past depressive episodes, age at onset, age, gender, ethnicity and body mass index) | Age was the strongest predictor of response to placebo, followed by duration of depression. Depressive symptom severity and anxiety symptom severity were among the top predictors of remission with Duloxetine and with SSRIs but not as strongly as age was for placebo. Number of past episodes, gender, and ethnicity were less important in all models, age at onset was moderately important in the model of remission with SSRIs but not the placebo or Duloxetine models. | Treated data as a single cohort pooling effects for each treatment across studies without adjusting for between study factors; no adjustment for study or random effects for study fitted, and no assessment of heterogeneity. No fully held-out test data, validation was internal only and did not involve cross-folding training data. Unclear methods of variable selection as separate from process of model building, also unclear on sensitivity analyses and model stability checks. AUC was only metric of model performance and not other important measures such as Brier scores, deviance, log-loss or others. Also no assessment of study attrition. |
| Steinert et al., 2014 (Steinert, Hofmann, Kruse, & Leichsenring, 2014) | Systematic Review | Naturalistic cohort studies with at least 3 year follow-up | K=12(N/A)  N=4009(N/A) | Community or general practice identified cases | Natural course and prognosis irrespective of treatment | Depressive symptom severity, previous episodes, comorbidities, diagnoses/subtypes of depression, social support, life events, childhood maltreatment, age of onset, educational attainment, and SES. | Owning a home and social support after a negative life-event were associated with recovery. Physical and sexual abuse in childhood as well as adult emotional abuse was associated with a lack of recovery, as was a personal or family history of depression. Personal history of depression (2 studies), onset age (1 study), dysthymia and double depression (1 study), baseline severity of depression (4 studies), and comorbidity (2 studies: anxiety disorder and personality disorders associated with long-term course in one study, personality disorders in the other study) were associated with poorer prognoses. Factors associated with a more favourable course were rare and only pointed out possible worthwhile future approaches. They comprised social support (1 study), a higher social and educational status (2 studies) as well as a higher onset age (1 study). | Mix of effects for recurrence, long-term course, treatment outcomes etc, difficult to isolate effect to prognosis after treatment. Range of sample sizes with no weighting of effects (range from n=33 to n=1996). Authors stated that attrition was not consistently reported and varied considerably across the reviewed studies. |
| Dodd et al., 2013 (Dodd, Berk, Kelin, Mancini, & Schacht, 2013) | IPD | Industry sponsored Phase III and IV RCTs of Duloxetine | K=15(15)  N=5627(5627) | Unclear | Response to a particular treatment (antidepressants), | Number of previous episodes | No main effect was found for the number of previous episodes, comparing three or more to less than three, and none to at least one. | Analyses were conducted with data treated as a cohort, no weighting or adjustment for study and no assessment of heterogeneity. No assessment of study attrition/ |
| Kampman & Poutanen, 2011 (Kampman & Poutanen, 2011) | Systematic Review and study-level meta-analysis | studies published 1991-2010 that used the temperament and character inventory | K=10(10)  N=938(938) | Non depressed adult community samples, some young adults, some specific populations e.g. school teachers | Natural course; prognosis irrespective of treatment | Types of temperament including harm-avoidance, reward dependence, novelty seeking, persistence, self-directedness, cooperativeness, and self-transcendence. | Harm avoidance temperament was associated with prognosis in clinical samples, others were not. | Mixture of clinical and non-clinical samples, some very small studies included (e.g. n=35). Used fixed not random effects models. Mixing of endpoints (from 6 weeks to 2-years) in same meta-analyses and no harmonisation across different measures. Makes sources of heterogeneity hard to interpret and main findings difficult to interpret too. |
| Fournier et al., 2010 (Fournier et al., 2010) | IPD | placebo-controlled RCTs | K=6(6)  N=718(718) | Outpatients | Response to a particular treatment (antidepressants), prescriptive test of interaction | Depressive symptom severity, | Baseline depressive symptom severity was associated with prognosis within those that received antidepressants and those that received placebo, there was also a significant interaction so higher severity patients responded better to antidepressants than placebo. Significant interaction between baseline depressive symptom severity and attrition. | Tests of interaction without within study step (falling foul of Fisher et al., 2017 guidelines) only 6 out of 23 eligible studies were included and no use of study-level data from the remaining 17 to consider effects. No control for missing data. Study attrition was high in 2 studies, particularly in the medication arm of one small study (34%). |
| Bower et al., 2013 (Bower et al., 2013) | IPD | RCTs reported since 2000 with n>50 | K=16(16)  N=2470(2470) | Community or primary care settings, included patients with depression and also those with mixed anxiety and depressive disorder | Response to a particular treatments (low intensity CBT), prescriptive test of interaction | Depressive symptom severity | Depressive symptom severity was related to outcome both with LI CBT and controls, there was moderation by severity so LICBT patients experienced better outcomes as severity increased vs control patients. | Only 55% of eligible studies/57% of eligible patients included so a number of potential biases may have affected results. Results may not be generalisable to patients diagnosed with depression due to more lenient inclusion criteria. Differential dropout rates between interventions could have led to systematic bias in moderator analysis. Used a crosswalk for BDI to CORE-OM or vice versa. Attrition assessed but not reported for each study. |
| Chekroud et al., 2016 (Chekroud et al., 2016) | Not Systematic Review but used IPD | N/A convenience sample | K=3(3)  N=4326(2234) | Primary care and psychiatric outpatient | Response to a particular treatments (antidepressants), model built with one treatment tested in other treatments without test of interaction | All baseline variables available. Top 25 used in final predictive models included: depressive symptom severity, history of antidepressant medication, history of prior episodes, comorbid anxiety symptoms, comorbid panic attacks, race/ethnicity, employment status and years of education. | Depressive symptom severity, employment status and years of education were among the top predictors of outcomes. Comorbid anxiety, history of antidepressant medication (Sertraline) and race/ethnicity also associated with outcome. | Convenience sample of data, machine-learning model used to find significant predictors but direction of effects difficult to determine with complex model. No details on the studies that would have been eligible but that were not included, no assessment of heterogeneity is provided and difficult interpreting reasons for differential model performance across study groups. Study attrition not assessed although last observation carried forward analysis for STAR*D participants is provided. |
| Weitz et al., 2015 (Weitz et al., 2015) | IPD | RCTs that randomised to CBT vs antidepressants | K=16(16)  N=1700(1700) | Not inpatients, otherwise fairly unclear. 3 studies included specific populations (patients with multiple sclerosis, women with low incomes, and women with infertility problems) | Response to a particular treatments (CBT and Antidepressants), with prescriptive test of interaction | Depressive symptom severity | Depressive symptom severity was associated with continuous symptom outcomes independent of treatment group, but not to response (50% reduction in symptoms) outcome, and no evidence was found for differential effect of CBT vs antidepressants. | Imputation conducted across the whole sample not within each study first. Used 1-stage meta-analyses with multi-level effects with individual effects on one level and study level effects at another level of the model. 1/3 of eligible studies did not provide data. Attrition is not assessed, risk of bias due to lack of intention-to-treat (ITT) analysis in studies is reported; four studies did not use an ITT but attrition rates are not reported in the review. |
| Karyotaki et al., 2017 (Karyotaki et al., 2017) | IPD | RCTs of internet guided low-intensity CBT | K=13(13)  N=3876(3876) | 7 of 13 recruited community participants - others unclear | Response to a particular treatment (internet based self-guided CBT), with prescriptive test of interaction | Depressive symptom severity, comorbid anxiety, age, sex, educational level, relationship status, employment status | Depressive symptom severity was associated with outcomes but no moderation by severity was found. None of the other factors were associated with outcomes but adherence moderated outcome between iCBT and controls. Analyses controlled for baseline depressive symptom severity. | A mix of 1-stage and 2-stage meta-analyses and of IPD and study-level analyses, which is perhaps unnecessary. Study focussed on moderation rather than prognostic effects, and had moderate to high heterogeneity that could not be well explained. Most were community samples rather than patient samples so generalisability to clinical settings/treated patients is limited. All studies reported as low risk of bias in all domain but study attrition not reported. |
| Driessen et al., 2010 (Driessen, Cuijpers, Hollon, & Dekker, 2010) | Study-level meta-analysis | RCTs | K=132(16)  N=10134(N/S) | Outpatients | Response to particular treatment (psychological therapies), prescriptive test of interaction | Depressive symptom severity | No evidence that pre-treatment severity predicted response to psychological treatment vs control condition; in a subset of studies with within study severity findings reported, psychological therapy was more effective with higher levels of severity | Findings are from meta-regression using mean severity as predictor of response and use prescriptive design to test treatment-type by severity interaction, could be considered to fall foul of appropriate tests of interactions in meta-analyses as per Fisher et al., 2017. Study attrition rates not reported. |
| Nakabayashi et al., 2018 (Nakabayashi, Hara, & Minami, 2018) | IPD | Placebo-controlled RCTs submitted to pharmaceuticals and medical devise agency in Japan. | K=5(5)  N=1898(1898) | Adults with MDD and no comorbidities, 2 studies excluded patients with treatment resistant depression. All patients were Japanese in 4 studies, and Japanese or Korean in 1 study. | Response to particular treatment (antidepressants), prescriptive test of interaction | Depressive symptom severity, history of antidepressant medication, age | Baseline depressive symptom severity was associated with prognosis within those that received antidepressants and those that received placebo. History of antidepressant medication and age were associated with response to antidepressants but not placebo, gender was not associated with response to either antidepressants or placebo. Age, and a history of past antidepressant medication moderated the effect of antidepressants compared to placebos. | Regression analyses performed one-step without effects calculated within trial or adjusted for allocation within each trial, and interactions were tested across studies treating data like a cohort (not following recommendations of Fisher et al., 2017). Dropout rates for the included studies were around 10-15 % for all conditions though in one study they were above 20% in each arm. No adjustments were made for baseline severity in assessment of other prognostic variables. |
| Gariepy et al., 2016 (Gariepy, Honkaniemi, & Quesnel-Vallee, 2016) | Systematic Review and study-level meta-analysis | Observational general population studies in "western countries" | K=36(36)  N= N/S | General population | Natural course | Social support | Most studies reported associations between social support and protection from depression, most evidence for emotional support followed by instrumental support. | Not treatment seeking sample. 31 studies included were of children or adolescents, 33 were of older adults. Most studies were cross-sectional (28 of 36 in general adult age group) so cannot rule out reverse causality. Only 5 studies rated high quality. Combined estimates from adjusted models in included studies so estimates adjusted for different variables making interpretation of heterogeneity complex. Nearly all studies used different social support measures, only 1/3 used previously validated measures, so comparisons complicated. No assessment of attrition. |
| Schoemaker et al., 2018 (Schoemaker, Kilian, Emsley, & Vingerhoets, 2018) | Meta-review with Qualitative Synthesis | SRs and study-level meta-analyses of placebo controlled RCTs | K=58(N/A)  N= N/S | Unclear | Response to particular treatment (placebo) | Depressive symptom severity, duration of illness, duration of episode, comorbid anxiety, comorbid health problems, age, gender, race/ethnicity, body mass index, trial design factors | Very weak evidence for a decrease in response to placebos with: 1) increased baseline severity, 2) increased duration of illness, 3) increased duration of current episode, 4) comorbid anxiety/somatization. Strong evidence for absence of effect on placebo response for age and very strong evidence for the same with sex. Weak evidence of absence of effect of race/ethnicity and comorbid physical health conditions, very weak evidence for absence of effect of concomitant medication. | Overlap in RCTs included in the review. Evidence for positive or negative associations all weak or very weak. Results regarding symptom severity come from 3 narrative meta-reviews of meta-analyses with 2 finding a weak effect and one finding no effect. No assessment of attrition within the reviewed reviews or primary studies. |
| Paykel, 1994 (Paykel, 1994) | Review, unclear if systematics | Unclear | K=29  N=N/S | Some outpatients, some general population, some psychiatric patients, generally unclear. | Natural course, and Irrespective of Treatment | Life events | Some evidence that those with life events prior to starting treatment might have poorer prognosis than those without life events. | Methods are somewhat unclear. In narrative review some studies showed effects and are compared to studies of prognosis in a different context, different setting and with very different samples making interpretation difficult. No quantitative synthesis and no comments on heterogeneity of findings, control for other factors, or reverse causality. No assessment of attrition. |
| Carter et al., 2012 (Carter et al., 2012) | Systematic Review | Studies published 1998-2008; adults, any study type, n>50. | K=76(N/A)  N=Unclear(N/A) | Unclear | Response to particular treatment (antidepressants) | Socio-demographics (age, gender, race/ethnicity, marital status, and SES), Clinical (depressive symptom severity, frequency and duration, comorbid anxiety, comorbid pain, other comorbidities, substance abuse), and Social support | Strong evidence for: baseline severity (14 studies); duration of depression (3 studies); social support (4 studies), SES (4 studies); comorbid pain (4 studies). Good evidence for prognostic associations with: age (10 studies) age at onset (3 studies), gender (9 studies), marital status (4 studies), comorbid anxiety (7 studies), and other comorbidities (6 studies). Some evidence for associations with: marital status (4 studies); number of past episodes (1 study) and substance abuse (1 study). Age (3 studies) and gender (1 study) were not associated with attrition. Ethnicity was associated with attrition (1 study) – Caucasians were less likely to dropout than non-Caucasians. | Mixture of reviews and primary studies, some double counting of effects. Effects of several factors claimed to have strong evidence later stated as inconclusive, particularly age and gender. Considerable heterogeneity, unclear of weighting in decisions about what counts as "strong evidence" vs "good evidence" etc. No harmonisation of data so mixed a number of different subtypes of anxiety, or comorbidities, of SES factors, and combined frequency of depressive episodes with duration of depression at baseline, hampering interpretation. |
| Sockol, 2018 (Sockol, 2018) | Systematic review and study-level meta-analysis | Studies of IPT in perinatal women, including RCTs, quasi-randomised trials, and open trials. | K=17(17)  N=790(790) | Unclear | Response to a particular treatment (IPT), prescriptive test of interaction | Depressive symptom severity, age, marital status, ethnicity | As the proportion of study participants that were married was increased the effect size of IPT on depressive symptoms increased. The converse was found when the proportion of 'minority' patients increased. Higher depressive symptom severity was associated with larger effect sizes when measuring change in symptoms pre-post treatment. Higher maternal age was associated with smaller effect sizes. | Of the 17 studies most had very small samples (e.g. n=6, 11, 11, 12, 13, 17, 18, 23, 32, 42, 48, 50, 50, 53). Main results regarding prognostic indicators had very high heterogeneity affecting interpretability of the results. Attrition ranged from 0-56% in studies, with a mean of 16%. No adjustments for baseline depressive symptom severity for other prognostic variables. |
| Cuijpers et al., 2018 (Cuijpers, Karyotaki, Reijnders, & Huibers, 2018) | Study-level meta-analysis | RCTs psychological treatments with any comparator. Acute treatment studies not relapse prevention. | K=256(256)  N=Unclear | Unclear | Response to a particular treatment (Psychological therapy), prescriptive test of interaction | Age, gender, ethnicity, depressive diagnosis (e.g. chronic, subthreshold) | Studies in students had higher effect sizes than studies of older adults. Studies of adults with general medical disorders had lower effect sizes than other studies. No difference in effect sizes based on gender, ethnicity, or severity of diagnosis. | Prognostic factors not tested specifically in the studies, meta-regression used to test differences where studies were of one particularly population subtype (e.g. students) and compared to another subtype (e.g. older adults). Generally high levels of heterogeneity. Attrition per study was not reported in the review but 50% of studies did not use an intention-to-treat analysis. |
| Cristea et al., 2019 (Cristea, Karyotaki, Hollon, Cuijpers, & Gentili, 2019) | Systematic review and study-level meta-analysis | RCTs of psychological therapy with biological markers measures pre-treatment | K=8(1 to 2)  N=Unclear | Unclear setting; Mostly adults with MDD but some with other mood disorders or just depressive symptoms, most studies in populations with somatic diseases | Response to a particular treatment (Psychological therapy), prescriptive test of interaction | Biomarkers - functional connectivity, brain metabolism and genetic polymorphisms. Others were examined but not as prognostic factors | One study found that functional connectivity between the subgenual cortex and prefrontal, insula and midbrain regions was associated with outcomes from CBT and antidepressants (Duloxetine, Escitalopram). Another study showed brain metabolism in 6 regions, most notably the right anterior insula was associated with outcome from CBT and Escitalopram. A third study found that those responding to CBT vs Venlafaxine showed increased metabolism in the inferior temporal cortex, and decreased metabolism in the posterior cingulate. No interaction effect was found between treatment and serotonin inhibitor polymorphisms. A study of the brain-derived neurotrophic factor polymorphism reported no prognostic or prescriptive association for the genotype under study although an interaction was found between the genotype and childhood adversity that was associated with prognosis across randomised groups. | Very few studies of prognostic association between biomarkers and outcome. Very difficult to interpret sources of heterogeneity and generalisability of findings questionable. Also overall 10 studies were rated as high risk of bias. 2 studies had high risk of bias due to incomplete data. Specific attrition rates were not reported in the review. |
| Ebrahim et al., 2012 (Ebrahim et al., 2012) | IPD | RCTs of CBT vs no treatment, usual care, or minimal treatment, that had disability benefit status as inclusion criterion | K=8(2) N=1502(227) | Unclear | Response to a particular treatment (CBT) | Disability benefit status | Tentative suggestion that effect size may have been higher in patients in receipt of disability benefit compared to those not in receipt of such benefits. | Most studies did not provide IPD. Only 34 people had exposure of interest in IPD, so difficult to interpret results. No adjustment for baseline depressive symptom severity. Attrition ranged from 4%-40% across the studies. No adjustment for baseline depressive symptom severity. |
| Lin et al., 2019 (Lin et al., 2019) | Systematic review and study-level meta-analysis | Studies of Venlafaxine with measures of CYP2D6 metaboliser status and pharmacokinetic outcomes | K=14(1 to 3), N=1035(12 to 571) | Included studies of healthy volunteers (K=5) as well as depressed samples (K=9). Settings unclear. | Response to a particular treatment (Venlafaxine) | Phenotypes of CYP2D6 gene | CYP2D6 phenotypes were not associated with response to Venlafaxine. | No control for type of study (health volunteers or patients) and majority of studies had very small sample sizes (e.g. 12, 14, 20); one study with more participants than 12 of the others combined, and most of the meta-analyses had only two or three studies so heterogeneity hard to interpret. 9 studies reported to not have clear reporting of attrition rates or gave no reasons for attrition. |
| Kloiber et al., 2013 (Stefan Kloiber et al., 2013) | Not SR but used IPD | N/A convenience sample | K=3(3), N=2256(2256) | 338 inpatients and 346 controls from the Munich Antidepressant Response Signature project, a case-control study. Also 672 patients from GENDEP and 980 from STAR*D | Response to a particular treatment (antidepressants) | In meta-analysis only polymorphisms of leptin gene, in single study also leptin mRNA expression and leptin serum levels. | In the meta-analyses no significant associations were found between polymorphisms of the leptin gene and antidepressant treatment outcomes. Such associations were found in the exploration and replication samples from MARS (single study). In the single study (MARS) lower leptin serum levels and reduced leptin mRNA expression were associated with poorer treatment outcomes independent of leptin genotype. | Only three studies included in convenience sample with no statement on the number of other studies that might have been eligible but were not approached for IPD. No details on the methods of meta-analysis or statements about heterogeneity are provided. No assessment of study attrition. No adjustment for baseline depressive symptom severity. |
| Morris et al., 2009 (B. H. Morris, Bylsma, & Rottenberg, 2009) | Systematic review | Prospective studies measuring the relationship between positive and negative emotionality and course of MDD | K=6 to 22(N/A), N=245 to 3553(N/A) | Adults aged 18-65 diagnosed with MDD. Setting unclear | Natural course | State and trait levels of negative and positive emotionality | Lower levels of positive emotionality were associated with poorer MDD course. Lower levels of state negative emotionality and higher levels of trait negative emotionality were associated with poorer MDD course. The associations in individual studies assessed were often present after controlling for baseline depressive symptom severity. | For state analyses combined a variety of ways or measuring emotionality (e.g. heart rate, skin conductance and self-report) and different measures of depression and different time intervals all in the same analysis. This makes interpretation and understanding sources of heterogeneity very difficult. Similar issues for trait analyses: combining resting EEG asymmetry with extraversion measures, combining treatment response with diagnostic status and relapse, at intervals of 2-36 months. Also combined some studies that included treatment with those in non-treated samples (some combined prognosis irrespective of treatment with natural state). No assessment of attrition or control for baseline depressive symptom severity. |
| Iovieno et al., 2011 (Iovieno, Tedeschini, Ameral, Rigatelli, & Papakostas, 2011) | Study-level meta-analysis | Placebo controlled-RCTs of antidepressants in patients with co-morbid long-term health conditions | K=212(190) without Axis-III inclusion criteria & 29(25) with Axis-III inclusion criteria  N=46900(46900) without Axis-III & 2338(unclear) with Axis-III | Unclear | Response to a particular treatment (antidepressants) | Comorbid long-term health conditions (“Axis-III” disorders in DSM) | Studies that specifically selected patients with Axis-III disorders comorbid to MDD had higher response rates to antidepressants compared to studies that did not use Axis-III comorbidity as a selection criteria. There was a non-significant trend towards the same effect in those randomised to pill placebos. Analyses adjusted for baseline symptom severity. | All included studies were efficacy trials so results may not be generalisable to wider MDD population. Combining results across studies with very different inclusion/exclusion criteria may make inferences invalid, and although studies for comparison did not have Axis-III conditions as an inclusion criterion it is not known what proportion of their participants had Axis-III conditions. No assessment of study attrition. |

* Note numbers do not correspond to total numbers of studies and participants assessed overall in each study, instead they represent numbers of studies (k) and participants (n) of relevance to this review.

## Details of search terms and search results for RCTs to form IPD dataset

**Supplementary Table 3.** Bibliographic database searches and results

| **Searches** | **Results** |
| --- | --- |
| **Cochrane CENTRAL Trial Register (searched on 1^st^ December 2020)** |  |
| 1. ("Depression" or "MDD" or "Unipolar" or "Depressive"):ti,ab,kw (Word variations have been searched) | 81260 |
| 2. (“RCT” or "controlled trial" or "randomized controlled trial" or "clinical trial"):ti,ab,kw (Word variations have been searched) | 1061566 |
| 3. ("CIS-R" or "Clinical Interview Schedule" or “Revised Clinical Interview Schedule” or “Clinical Interview Schedule Revised”):ti,ab,kw (Word variations have been searched) | 63 |
| 4. #1 and #2 and #3 | **49** |
| **Embase 1947 to 2020 November 30** |  |
| 1. (depression or Depressive disorder or Major depression or Unipolar depression or MDD).mp. | 7288455 |
| 2. exp controlled clinical trial/ or exp "randomized controlled trial (topic)"/ or exp "clinical trial"/ | 1757626 |
| 3. ("Clinical Interview Schedule" or "CIS-R" or "CISR" or "Revised clinical interview schedule" or "clinical interview schedule revised").af. | 857 |
| 4. 1 and 2 and 3 | **33** |
| **International Pharmaceutical Abstracts 1970 to October 2020** |  |
| 1. (depression or Depressive disorder or Major depression or Unipolar depression or MDD).mp. | 10409 |
| 2. (RCT or controlled trial or randomized controlled trial or clinical trial).mp. | 15679 |
| 3. ("Clinical Interview Schedule" or "CIS-R" or "CISR" or "Revised clinical interview schedule" or "clinical interview schedule revised").af. | 3 |
| 4. 1 and 2 and 3 | **0** |
| **Ovid MEDLINE 1946 to December 01 2020** |  |
| 1. exp major depression/ or exp "depression (emotion)"/ | 122064 |
| 2. exp Depressive Disorder, Major/ | 30626 |
| 3. exp Depressive Disorder, Major/ or exp Depressive Disorder/ or exp Depression/ | 219973 |
| 4. 1 or 2 or 3 | 219973 |
| 5. exp controlled clinical trial/ or exp "randomized controlled trial (topic)"/ | 608159 |
| 6. ("Clinical Interview Schedule" or "CIS-R" or "CISR" or "Revised clinical interview schedule" or "clinical interview schedule revised").af. | 621 |
| 7. 4 and 5 and 6 | **21** |
| **PsycINFO 1806 to November Week 4 2020** |  |
| 1. exp major depression/ or exp "depression (emotion)"/ | 158704 |
| 2. (depression or Depressive disorder or Major depression or Unipolar depression or MDD).mp. | 344487 |
| 3. 1 or 2 | 344715 |
| 4. exp "randomized controlled trial (topic)"/ or exp "clinical trial"/ or exp "controlled trial"/ or exp "randomized clinical trial"/ | 12545 |
| 5. (RCT or controlled trial or randomized controlled trial or clinical trial).mp. | 43921 |
| 6. 4 or 5 | 50453 |
| 7. ("Clinical Interview Schedule" or "CIS-R" or "CISR" or "Revised clinical interview schedule" or "clinical interview schedule revised").af. | 1223 |
| 8. 3 and 6 and 7 | **47** |
| **Cochrane CENTRAL Trial Register (searched on 1^st^ December 2020)** |  |
| 1. ("Depression" or "MDD" or "Unipolar" or "Depressive"):ti,ab,kw (Word variations have been searched) | 81260 |
| 2. (“RCT” or "controlled trial" or "randomized controlled trial" or "clinical trial"):ti,ab,kw (Word variations have been searched) | 1061566 |
| 3. ("CIS-R" or "Clinical Interview Schedule" or “Revised Clinical Interview Schedule” or “Clinical Interview Schedule Revised”):ti,ab,kw (Word variations have been searched) | 63 |

### Identification and selection of studies

A single reviewer (JB) screened titles and abstracts of potentially eligible studies, these were then read in full and judged against inclusion/exclusion criteria by two reviewers (JB and GL) with consultation with a third (SP) to resolve any uncertainties by consensus. This resulted in two studies previously included in the Dep-GP (Kendrick et al., 2005; Proudfoot et al., 2003) and discussed in the registration and protocol documents for this review, later being excluded as they did not meet our inclusion criteria; they were not studies of patients with depression or who were seeking help for depressive symptoms.

#### Data Extraction

Raw data were extracted for each study participant on all variables in Table 2. Data were cleaned one study at a time, independently by two reviewers (JB and RS), and crosschecked with publications and via liaison with chief investigators for each study. Issues were resolved by consensus between four reviewers (JB, RS, GL and SP).

#### Secondary outcomes

1. Remission on the primary depression measure in each study at 3-4 months (see Supplementary Table 4 for how this was defined).
2. Depressive symptoms at 6-8 months, as above, captured with i) the z-score calculated using the mean and standard deviation for the scores at 3-4 months, and ii) the logarithm of scores at 6-8 months.

#### Secondary and sensitivity analyses

Sensitivity analyses were planned if there was considerable heterogeneity,(Higgins & Green, 2011) either from inspection of the forest plots or if I^2^ was 75% or above for models 1-4, or 50% or above for any variables included in the final models, removing the study contributing most to the heterogeneity.

### Risk of Bias

Risk of bias assessments were conducted using the Quality in Prognosis Studies (QUIPS) (Hayden, Windt, Cartwright, Côté, & Bombardier, 2013) and the quality of evidence for each prognostic indicator was assessed using the GRADE framework (Guyatt et al., 2008). Two independent reviewers conducted the risk of bias assessments (JB & RS).

### Patient and Public Involvement

Service user advisory groups of two primary care mental health services in central London and the expert service user researchers of the Service User Research Forum (SURF) were consulted for advice on the design, conduct, and dissemination of this study.

## Details of additions, deviations and changes to protocols

We have registered the process of finding studies and the research questions for this study in PROSPERO (CRD42019129512) and produced a protocol paper that was amended twice. Below we will explain the amendments made and the process of finding studies and forming the dataset for this study.

We started this project with one of the senior investigators (GL) in possession of individual patient data from two studies for which he was the chief investigator, and a third study that he was in the process of conducting. We ran scoping searches, noted that the CIS-R was the most commonly used comprehensive measures of depressive and anxiety symptoms and disorders in RCTs of depression set in primary care, and refined our scoping searches to look for studies that used the CIS-R. That author (GL) was a co-investigator on a number of other trials that used the CIS-R and we made contact with the chief investigators of those studies to ask for in-principle agreement to access IPD from their trials and then applied for funding for this project. Once funding was in place we registered our project on PROSPERO, at that point we had run two rounds of searches (scoping searches and one set to inform our funding application), and we had obtained IPD data from four studies. We refined our searches by including two other databases and reaching out to experts for missed studies, this helped us find further studies. We invited the chief investigators from each of those studies to join the project. We began to collect some further IPD from the studies that had agreed to take part. We then wrote up a protocol paper with information of what we would do with those IPD data once the dataset was complete. We ran further searches and found one more study just before initially submitting the protocol paper. It was a protracted process to gain IPD from that study but the idea was that the Dep-GP IPD dataset would be formed from all of those studies we had found. The Protocol paper was peer-reviewed and we amended it post-review to give more details about this process. The protocol was then accepted for publication. It was amended once more when we decided that our choice of an I^2^ threshold for considering problematic heterogeneity was too high, we dropped it from 80% to 75% for all models and to 50% for the final models, in line with recommendations from Cochrane. We ran the final searches for studies meeting our inclusion criteria a before submitting this manuscript for publication and found no new studies meeting our criteria.

Our protocol paper provides information about all data we sought to extract from the included studies and all outcomes of interest. For the present study we were particularly interested in factors associated with severity of a patient’s clinical presentation, and so limited analyses to such factors. Future studies using these data will consider the prognostic associations between other non-severity related factors at baseline and prognosis. Further, for this study we amended our inclusion criteria slightly to exclude studies that recruited those with either depression or an anxiety disorder at baseline because this was a study specifically about depression and the impact of comorbid anxiety as an indicator of prognosis was a key question for the study. There were two changes to the statistical analysis plan that should be noted: 1) we did not include attrition as an outcome for the present study; and 2) we added further analyses to consider the impact of the prognostic variables on the variance in outcome that is explained.

### Secondary Outcomes

#### Remission at 3-4 months post-baseline

There were very few differences in the factors associated with remission and those associated with the primary outcomes, see Supplementary Table 7.

#### Depressive symptoms at 6-8 months post-baseline

Seven studies had an end-point at 6-8 months post-baseline. All of the severity factors associated with the primary outcome at 3-4 months were also associated with the outcome at 6-8 months post-baseline, see Supplementary Table 8.

### Sensitivity Analyses

Removing studies due to heterogeneity resulted in very small differences in magnitudes of effect and had no impact on the direction of effects or conclusions that might be drawn from the primary analyses, see Supplementary Table 9. Details of further sensitivity analyses are given in the Supplementary Materials.

### Calculating variance explained in the primary outcome

In order to calculate the variance explained in the depressive symptom scale scores at 3-4 months and the increases in variance explained when adding each ‘disorder characteristic’ included in the final models, we ran two-stage random effects meta-analyses as described in the main manuscript, using “admetan” in Stata. We initially included only the depressive symptom severity score at baseline, random treatment allocation in each study, age and gender, in the model. This was calculated in each imputed dataset for each study, then the mean adjusted r^2^ was calculated by pooling at the imputed dataset level first, then averaging again at the study level. Once this was estimated for a model containing the above variables, each time a new variable was added the adjusted r^2^ was calculated again using the same method so that the contribution of each ‘disorder characteristic’ to the overall variance explained in the outcome could be considered.

**Supplementary Table 4.** Measures used across the studies of the Dep-GP IPD database

| **Measure** | **Details** | **Scores and Cut-offs for Remission** |
| --- | --- | --- |
| The CIS-R(Lewis, Pelosi, Araya, & Dunn, 1992) | Consists of 14 symptom subsections scored 0-4 covering core features of depression, depressive thoughts (scored 0-5), fatigue, concentration/forgetfulness, and sleep, generalized anxiety, worry, irritability, obsessions, compulsions, health anxiety, somatic concerns, phobic anxiety (split into agoraphobia, social phobia, and specific phobia), and panic. A final section measures general health, impairment and weight change. | The total score ranges from 0-57 with a cut-off of ≥12 used to indicate likely common mental disorder, primary and secondary diagnoses using ICD-10 criteria are given as are binary indictors of diagnosis for all the disorders assessed. The duration of each type of problem is also assessed for the present episode (or subsyndromal episode) unto the point of completing the CIS-R. Duration items are measured in five categories: 1) less than two weeks; 2) between two weeks and six months; 3) between six months and one year; 4) between one and two years; and 5) more than two years. |
| Beck Depression Inventory 2^nd^ Edition (BDI-II)(Beck, Steer, & Brown, 1996) | Consists of 21 items to assess depressive symptoms, each item is scored 0-3. | There is a maximum score obtainable of 63, and a cut-off of ≥10 is used indicate significant symptoms of depression, scores of <10 are therefore used to indicate remission in those that were previously depressed/scored ≥10. |
| Patient Health Questionnaire 9-item version (PHQ-9)(Kroenke, Spitzer, & Williams, 2001) | This is a depression screening measure, with respondents asked to rate how often they have been bothered by each of the nine symptom items over the preceding two weeks. Each item is scored 0-3 | There is a maximum score of 27 with a cut-off of ≥10 is used to indicate “caseness” for depression, a score of 9 or below for those that were previously depressed is therefore considered to indicate remission. |
| Hospital Anxiety and Depression Scale (HADS)(Zigmond & Snaith, 1983) | Measures symptoms on two subscales, depression and anxiety, each with 7 items scored 0-3. Respondents are asked to endorse a statement relating to frequency or severity of problems in each symptom domain over the preceding 7 days | A total score of 21 is obtainable on each subscale, with a cut-off for caseness on the depression subscale of ≥8. Scores <8 are therefore used to indicate remission. |
| General Health Questionnaire (12-item version) (GHQ-12)(Goldberg, 1992) | Consists of 12 items related to present and recent health over the “few weeks” prior to completion. Each item is related to depression or generalised anxiety, they are scored 0-0-1-1 for the four response options. | A cut-off of ≥2 is used to indicate the likely presence of common mental disorder, and so scores of <2 for those formally scoring above this would be considered to indicate remission. |
| Edinburgh Postnatal Depression Scale (EPDS)(J. L. Cox, Holden, & Sagovsky, 1987) | This measures symptoms of depression among women in the post-natal period. It consists of 10 items relating to symptoms of depression, each one is rated in relation to feelings over the week prior to completion. Each item is scores 0-3. | The maximum obtainable score is 30, with scores of ≥13 are indicative of a depressive episode, and scores of <13 indicative of remission among the formally depressed. |
| Generalised Anxiety Disorder Scale, 7-item version (GAD-7)(Spitzer, Kroenke, Williams, & Löwe, 2006) | This measures symptoms of generalised anxiety with the same scaling and structure of questions as used in the PHQ-9. | A maximum score of 21 is obtainable across the 7 items. A cut-off of ≥8 is used to determine ‘caseness’ for GAD. |
| Social Support Scale - adapted for use in Adult Psychiatric Morbidity Surveys from the Health and Lifestyles Survey.(B. D. Cox et al., 1987) | An 8-item instrument, the first seven items of which come from the Health and Lifestyles Survey assessing the degree to which participants rated the social support of their friends and family in each of the following domains: 1) being accepted for who one is; 2) feeling cared about; 3) feeling loved; 4) feeling important to them; 5) being able to rely on them; 6) feeling well supported and encouraged by them; 7) being made to feel happy by them; and 8) feeling able to talk to them whenever one might like. Items are scored 1-3, with total scores ranging from 8-24; higher scores indicate higher levels of perceived social support. The authors of the Health and Lifestyles Survey suggested the maximum score for social support (which was 21 on that scale) indicated ‘no lack of social support’ scores between 18-20 indicated a ‘moderate lack of social support’ and scores of 17 or below indicated a ‘severe lack of social support’. | N/A |
| Life events: the Social Readjustment Rating Scale (Holmes & Rahe, 1967) | Participants are asked to say yes/no to whether they have suffered any of eight events within the last six months e.g. a death/bereavement; being physically attacked/injured; or going through a divorce/separation. Each item is scored yes (1) or no (0) and the total score is the sum of all the items. | N/A |
| Alcohol use: the alcohol use disorder identification test primary care version (AUDIT-PC).(Piccinelli et al., 1997) | Used to assess alcohol misuse, this includes five items scored 0-4. A cut-off of ≥5 indicates hazardous alcohol use that may be harmful to one’s health | N/A |
| Health related quality of life: EQ-5D-3L & EQ-5D-5L.(Herdman et al., 2011) | The EQ-5D is a generic measure of health status in five domains – mobility; self-care; usual activities; pain/discomfort; and anxiety/depression. Each domain in the 3L version has three response categories ranging from no problem present (1) to extreme problems in the given domain (3), the 5L version has five response options ranging from “I have no problems…” (1) to “I am unable to…” or “I have/am extreme/extremely…” (5). A total score is derived from summing the score on the five items with higher scores indicating more severe health problems than lower scores. A cross-walk of scores from the 3L and 5L versions will be used to derive a continuous index score representing the EQ-5D total score in the present study (Hout, Janssen, Feng, Kohlmann, & Busschbach, 2012). | N/A |
| CIS-R was used in all 12 studies, for depression subscale scores and durations n=5686, for anxiety scores n=5415, for anxiety durations and individual diagnoses n=5088 . BDI-II was used in 6 studies (COBALT, GENPOD, IPCRESS, MIR, PANDA, & TREAD), n=2858 ; PHQ-9 was used in 6 studies (CADET, COBALT, HEALTHLINES, MIR, PANDA, & REEACT) n=3416 ; HADS was used in two studies (AHEAD & GENPOD) n=925; GHQ was used in ITAS only n =796; EPDS was used in RESPOND n=220 ; GAD-7 was used in 5 studies (CADET, COBALT, HEALTHLINES, MIR & PANDA) n=2110; the Social Support Scale was used in 6 studies (COBALT, GENPOD, IPCRESS, MIR, PANDA, & TREAD) n =2858; the Life Events, Social Readjustment Rating Scale was used in 7 studies (COBALT, GENPOD, IPCRESS, ITAS, MIR, PANDA, & TREAD) n=3656; the AUDIT-PC was used in 6 studies (COBALT, GENPOD, IPCRESS, MIR, PANDA, & TREAD) n=3028 ; EQ-5D was used in 8 studies (AHEAD, CADET, HEALTHLIENS, IPCRESS, MIR, PANDA, REEACT, & TREAD) n=3931. | | |

## Ethical Approvals and Trial Registrations details for studies included in Dep-GP IPD dataset

**Supplementary Table 5.** Ethical approval and Trial Registration details of the studies included in the Dep-GP IPD database

| **Study** | **Ethical Approvals** | **Trial Registration details** |
| --- | --- | --- |
| AHEAD | South West Multicentre Ethics Committee and ethics committees covering Hampshire, East Dorset, Wiltshire, West Sussex and South West Surrey | ISRCTN14453847; https://doi.org/10.1186/ISRCTN14453847 |
| CADET | Granted by NHS Health Research Authority & NRES Committee South West (NRES/07/H1208/60) | ISRCTN32829227; https://doi.org/10.1186/ISRCTN32829227 |
| COBALT | Approvals were granted by West Midlands Research Ethics Committee (NRES/07/H1208/60) and research governance approval was obtained from the local Primary Care Trusts/Health Boards | ISRCTN38231611; https://doi.org/10.1186/ISRCTN38231611 |
| GENPOD | The South West Research Ethics Committee granted approval (MREC 02/6/076) and the Bristol, Manchester and Newcastle Primary Care NHS Trusts granted research governance approval. | ISRCTN31345163; https://doi.org/10.1186/ISRCTN31345163 |
| HEALTHLINES | Approval was granted by the National Research Ethics Service Committee South West–Frenchay (Reference 12/SW/0009) | ISRCTN14172341; https://doi.org/10.1186/ISRCTN14172341 |
| IPCRESS | Approval granted by Royal Free and Hampstead Research Ethics Committee, reference number 05/Q0501/18 | ISRCTN45444578; https://doi.org/10.1186/ISRCTN45444578 |
| ITAS | Bro Taf Health Authority and United Bristol Healthcare Trust Local Research Ethics Committee | ISRCTN57116180; https://doi.org/10.1186/ISRCTN57116180 |
| MIR | Approvals were granted by South East Wales Research Ethics Committee Panel C (ref: 12/WA/0353); Bristol Clinical Commissioning Group (CCG), and other CCGs provided research governance assurance. | ISRCTN06653773; https://doi.org/10.1186/ISRCTN06653773 |
| PANDA | The Bristol Research Ethics Committee Centre granted ethics approval (12/SW/0267). | ISRCTN84544741; https://doi.org/10.1186/ISRCTN84544741 |
| REEACT | The Leeds (East) research ethics committee granted approval (08/H1306/77). | ISRCTN91947481; https://doi.org/10.1186/ISRCTN91947481 |
| RESPOND | Approvals were granted by the Scotland A Multi-centre Research Ethics Committee (MREC; reference number MREC/03/0/127) and site-specific approval was obtained from 10 relevant local ethics committees and 10 primary care trusts (PCTs) | ISRCTN16479417; https://doi.org/10.1186/ISRCTN16479417 |
| TREAD | Approvals were granted by West Midlands multicentre research ethics committee (MREC 05/MRE07/42), and research governance approval was given by the relevant local National Health Service primary care trusts | ISRCTN16900744; https://doi.org/10.1186/ISRCTN16900744 |

### Data Integrity Checks

Integrity of all baseline and endpoint data for each study were checked with the study team and against details published about each study. The numbers of participants included in the Dep-GP for some studies is very slightly different than those in the published articles about the individual studies. This is because a very small number of cases were removed from Dep-GP if they had missing data on over 75% of the variables at baseline or were missing all CIS-R variables, this resulted in two patients being removed from IPCRESS and one from PANDA. For CADET 54 participants withdrew after the study was completed so their data were not made available, and for ITAS there were complete data for 36 more participants than reported in the publications about that study.

### Quality assessments and Risk of Bias

Two reviewers independently judged the risk of bias in each study to be low in most domain, although half of the studies were judged as moderate risk of bias due to attrition. Based on the GRADE framework we considered the quality of evidence in regards each prognostic indicator to be high, see Supplementary Table 5; interrater reliability: (Cohen’s Kappa) k=0.98 for QUIPS and k=1.00 for GRADE.

**Supplementary Table 6**. QUIPS risk of bias ratings

| **Study** | **Study Participation** | **Study Attrition** | **Prognostic Factor Measurement** | **Outcome Measurement** | **Study Confounding** | **Statistical Analysis and Reporting** |
| --- | --- | --- | --- | --- | --- | --- |
| AHEAD | Low | Moderate | Low | Low | Low | Low |
| CADET | Low | Moderate | Low | Low | Low | Low |
| COBALT | Low | Low | Low | Moderate | Low | Low |
| GENPOD | Low | Low | Low | Low | Low | Low |
| HEALTHLINES | Low | Moderate | Low | Low | Low | Low |
| IPCRESS | Low | High | Low | Low | Low | Low |
| ITAS | Low | Low | Low | Low | Low | Low |
| MIR | Low | Moderate | Low | Low | Low | Low |
| PANDA | Low | Low | Low | Low | Low | Low |
| REEACT | Low | Moderate | Low | Low | Low | Low |
| RESPOND | Low | Moderate | Low | Low | Low | Low |
| TREAD | Low | Low | Low | Low | Low | Low |

**Supplementary Table 7.** GRADE quality rating for each evidence for each type prognostic factor assessed

|  | **Prognostic Indicator** | | | | | | | | | |
| --- | --- | --- | --- | --- | --- | --- | --- | --- | --- | --- |
| **Study** | **Depressive symptom severity** | **CIS-R score items** | **Depression Duration** | **CIS-R duration items** | **Comorbid Diagnoses** | **Number of comorbid diagnoses** | **Functional Impairment** | **History of Depression** | **Past ADM use** | **Past treatment for depression** |
| AHEAD | High | High | N/A | N/A | N/A | N/A | N/A | N/A | N/A | N/A |
| CADET | High | High | High | High | High | High | High | High | High | High |
| COBALT | High | High | High | High | High | High | High | High | N/A | N/A |
| GENPOD | High | High | High | High | High | High | High | High | High | High |
| HEALTHLINES | High | High | High | High | High | High | High | High | High | High |
| IPCRESS | High | High | High | High | High | High | High | High | High | High |
| ITAS | High | High | High | High | High | High | High | High | High | High |
| MIR | High | High | High | High | High | High | High | High | High | High |
| PANDA | High | High | High | High | High | High | High | High | High | High |
| REEACT | High | High | High | High | High | High | High | High | High | High |
| RESPOND | High | High | High | High | High | High | High | High | High | High |
| TREAD | High | High | High | High | High | High | High | High | High | High |
| **Overall** | **High** | **High** | **High** | **High** | **High** | **High** | **High** | **High** | **High** | **High** |

## Additional Details of Data Analyses

#### Software & packages

Stata SE 15(StataCorp LP., 2017): ipdmetan(Fisher, 2015), mvmeta(White, 2011), MICE(Royston, 2009), mi impute pmm(T. P. Morris, White, & Royston, 2014) packages.

#### Information on Dealing with Missing data

Missing data were imputed using multiple imputation with chained equations (MICE) in [Stata](https://www.stata.com/) 15.0. This approach uses regression models to impute missing values. A number of imputed datasets (here we used 50) are produced to reflect the uncertainty/variability in the imputation process. Data not reasonably able to be log transformed to meet normality assumptions, were imputed using predictive mean matching (PMM) via a k-nearest neighbours approach as it is considered to be more appropriate for non-normal continuous variables(Horton & Lipsitz, 2001), here we used k=10. Linear regression was used for approximately normally distributed continuous variables, logistic regression models for binary variables, and ordinal and multinomial regression models for ordered and unordered categorical variables respectively. All imputation models were built using data on baseline and outcome variables following conventions (Royston & White, 2011). Only variables with less than 50% missing data were imputed (see Supplementary Table 3 for degrees of missing by variable). All imputation models were run to produce 50 imputed datasets. If the primary analysis showed that results differed considerably when studies with systematically missing baseline data were included/excluded from the meta-analytic models, then a separate imputation approach would have been taken, to impute these systematically missing data using multiple imputation with multilevel random effects for study (Resche-Rigon, White, Bartlett, Peters, & Thompson, 2013).

#### Additional Sensitivity Analyses

Further to the sensitivity analyses described in the main manuscript additional analyses were planned if any studies had moderate or high risks of bias, or offered a low quality of evidence for the effects investigated (see Risk of Bias section).

Further analyses were conducted using the endpoint at 6-8 months in bivariate meta-analyses including the study that did not have an endpoint in the 3-4 month post-baseline time-period using the ‘mvmeta’ package in Stata.(White, 2011) The impact of variables that could not be imputed as they were not collected in any one of the Dep-GP studies was assessed by comparing results of meta-analyses with and without studies systematically missing any potential ‘disorder characteristic’. The two above sensitivity analyses were initially run to assess the association with prognosis of baseline depressive symptom severity adjusted for the specified covariates. If it were found that either of the above led to considerable variation in the results then the bivariate meta-analytic method was planned to be similarly used in the analyses of the other potential prognostic factors, or the systematically missing variables imputed and all analyses run over those data, accordingly. In addition, all analyses were also run on observed, un-imputed data to consider the impact of imputation.

Additional sensitivity analyses were conducted using all 11 studies that had an endpoint at 3-4 months, post-baseline, to build the final models of outcomes using only those variables available in all 11 studies. In order to include a measure of anxiety symptom severity, as the total of the anxiety subscales from CIS-R was not available in two studies (AHEAD & HEALTHLINES), the z-score of anxiety symptoms on all measures of anxiety used in the studies including the HADS, GAD-7 and CIS-R anxiety subscales was calculated in the same way as depressive symptom severity, using the mean and standard deviation of each symptom measure across all studies at baseline.

## Additional Results of Sensitivity Analyses

In sensitivity analyses using variables available in all studies, the ‘disorder characteristics’ that were associated with prognosis in the final models were the duration of depression, anxiety symptom severity, and a history of antidepressant treatment, all independent of treatment, depressive symptom severity, covariates, and each other, see Supplementary Tables 7 and 8, and Figure 3. As with the primary analyses, symptom severity was most strongly associated with prognosis, with each standard deviation increase at baseline associated with approximately 26% higher depressive symptom scale scores at 3-4 months post-baseline, see Supplementary Table 7. Again similarly to the primary analyses, the association between a history of antidepressant use and prognosis was significant when using the z-score outcome but not with the log outcome, however again it contributed to increases in the amount of variance explained with both outcomes, see Supplementary Table 8. Overall, the amount of variance explained with these four factors (three depressive ‘disorder characteristics’ and depressive symptom severity) was marginally lower than with the five factors from the primary analyses (24% for the z-score outcome and 18% for the log outcome; compared to 27% and 21% respectively in the primary analyses). Heterogeneity was somewhat higher with the factors assessed in all studies than in the primary analyses (see Supplementary Figure 1), particularly regarding the anxiety symptom severity variable, further sensitivity analyses removing studies to reduce the heterogeneity in this and other effects are presented in Table 11.

In addition, there were only very small differences in the magnitude (0.02 of a standard deviation in effect) of association of depressive symptom severity and prognosis when comparing a univariate meta-analysis of the z-score outcome at 3-4 months with a bivariate meta-analysis using both the 3-4 and 6-8 months z-score outcomes. There was an even smaller degree of difference (0.01 of a standard deviation) in associations when including or excluding the two studies that were systematically missing many ‘disorder characteristics’, and a similarly small difference in associations when using observed data compared to the main analyses using imputed data.

**Supplementary Table 8.** Difference in odds of remission at 3-4 months post-baseline per unit increase in baseline prognostic indicators.

|  | **Adjusted for Treatment, Age and Gender^ρ^** | | **Additionally adjusted for depressive symptom severity*** | | **Additionally adjusted for employment status and/or marital status ^‡^** | |
| --- | --- | --- | --- | --- | --- | --- |
| **Prognostic Indicator** | **OR(95%CI)** | **I^2^** | **OR(95%CI)** | **I^2^** | **OR(95%CI)** | **I^2^** |
| Depressive symptom severity | 0.49(0.44 to 0.54) | 39 | 0.49(0.44 to 0.54) | 39 |  |  |
| CIS-R Total Score | 0.94(0.93 to 0.95) | 31 | 0.97(0.96 to 0.98) | 18 |  |  |
| Depressive Subscales Total^‡1^ | 0.86(0.83 to 0.89) | 50 | 0.94(0.91 to 0.96) | 24 | 0.95(0.92 to 0.98) | 29 |
| Anxiety Subscales Total^‡2^ | 0.94(0.92 to 0.96) | 67 | 0.98(0.96 to 0.99) | 42 | 0.98(0.96 to 0.99) | 38 |
| Compulsions Score | 0.81(0.75 to 0.88) | 49 | 0.91(0.84 to 0.99) | 48 |  |  |
| Compulsions Duration^‡1^ | 0.91(0.87 to 0.95) | 25 | 0.96(0.92 to 1.00) | 16 | 0.96(0.92 to 1.00) | 3 |
| Concentration Score | 0.79(0.72 to 0.86) | 63 | 0.94(0.89 to 1.00) | 0 |  |  |
| Concentration Duration | 0.82(0.78 to 0.87) | 33 | 0.90(0.86 to 0.95) | 8 |  |  |
| Depression Score^‡3^ | 0.73(0.66 to 0.79) | 52 | 0.93(0.85 to 1.01) | 38 | 0.95(0.86 to 1.06) | 44 |
| Depressive Thoughts Score^‡1^ | 0.71(0.67 to 0.76) | 15 | 0.87(0.82 to 0.93) | 0 | 0.89(0.83 to 0.95) | 0 |
| Depression Duration^‡1^ | 0.78(0.73 to 0.84) | 40 | 0.83(0.77 to 0.90) | 41 | 0.85(0.79 to 0.92) | 39 |
| Fatigue Score^‡1^ | 0.85(0.76 to 0.96) | 64 | 0.93(0.84 to 1.02) | 36 | 0.94(0.86 to 1.03) | 22 |
| Fatigue Duration | 0.82(0.77 to 0.86) | 0 | 0.86(0.81 to 0.91) | 0 |  |  |
| Generalised Anxiety Score^‡1^ | 0.86(0.81 to 0.90) | 29 | 0.96(0.92 to 1.01) | 0 | 0.96(0.91 to 1.01) | 0 |
| Generalised Anxiety Duration | 0.89(0.86 to 0.93) | 0 | 0.93(0.89 to 0.97) | 0 |  |  |
| Health Anxiety Score^‡1^ | 0.80(0.75 to 0.85) | 23 | 0.90(0.85 to 0.96) | 2 | 0.93(0.87 to 0.99) | 3 |
| Health Anxiety Duration^‡3^ | 0.89(0.85 to 0.92) | 16 | 0.94(0.90 to 0.97) | 0 | 0.95(0.91 to 0.99) | 0 |
| Irritability Score^‡3^ | 0.87(0.83 to 0.92) | 0 | 1.00(0.94 to 1.05) | 0 | 0.98(0.93 to 1.04) | 0 |
| Irritability Duration^‡2^ | 0.88(0.83 to 0.92) | 29 | 0.92(0.87 to 0.97) | 20 | 0.92(0.87 to 0.97) | 20 |
| Obsessions Score | 0.91(0.86 to 0.97) | 54 | 0.98(0.92 to 1.04) | 46 |  |  |
| Obsessions Duration | 0.93(0.88 to 0.97) | 38 | 0.98(0.93 to 1.02) | 36 |  |  |
| Panic Score^‡2^ | 0.82(0.74 to 0.9) | 65 | 0.92(0.85 to 1.00) | 44 | 0.93(0.85 to 1.02) | 47 |
| Panic Duration^‡1^ | 0.85(0.81 to 0.90) | 26 | 0.92(0.88 to 0.96) | 0 | 0.93(0.89 to 0.97) | 0 |
| Phobias Score^‡1^ | 0.78(0.71 to 0.86) | 63 | 0.89(0.82 to 0.95) | 33 | 0.91(0.86 to 0.97) | 2 |
| Phobias Duration^‡3^ | 0.88(0.83 to 0.93) | 63 | 0.94(0.90 to 0.98) | 29 | 0.95(0.91 to 0.99) | 25 |
| Sleep Score^‡3^ | 0.81(0.77 to 0.85) | 48 | 0.92(0.87 to 0.97) | 38 | 0.94(0.88 to 1.00) | 49 |
| Sleep Duration^‡1^ | 0.84(0.81 to 0.88) | 5 | 0.89(0.85 to 0.93) | 4 | 0.89(0.86 to 0.93) | 0 |
| Somatic Score^‡3^ | 0.88(0.82 to 0.94) | 53 | 0.95(0.89 to 1.01) | 28 | 0.96(0.91 to 1.02) | 13 |
| Somatic Duration^‡3^ | 0.88(0.85 to 0.91) | 0 | 0.92(0.89 to 0.96) | 0 | 0.93(0.89 to 0.97) | 0 |
| Worry Score^‡1^ | 0.83(0.79 to 0.87) | 0 | 0.96(0.91 to 1.01) | 0 | 0.96(0.91 to 1.02) | 0 |
| Worry Duration | 0.85(0.81 to 0.89) | 0 | 0.89(0.85 to 0.94) | 0 |  |  |
| Average Duration of Anxiety^‡3^ | 0.65(0.58 to 0.74) | 58 | 0.78(0.70 to 0.86) | 36 | 0.79(0.71 to 0.88) | 30 |
| Number of Comorbid CMDs^‡3^ | 0.72(0.61 to 0.83) | 79 | 0.89(0.80 to 0.99) | 47 | 0.89(0.8 to 1.00) | 49 |
| Agoraphobia^‡1^ | 0.56(0.45 to 0.70) | 0 | 0.76(0.60 to 0.96) | 0 | 0.82(0.65 to 1.04) | 0 |
| CFS^‡2^ | 0.61(0.49 to 0.76) | 52 | 0.86(0.73 to 1.00) | 0 | 0.83(0.71 to 0.98) | 2 |
| GAD | 0.67(0.58 to 0.77) | 26 | 0.90(0.78 to 1.04) | 0 |  |  |
| MADD | 1.45(1.26 to 1.67) | 11 | 1.12(0.96 to 1.30) | 0 |  |  |
| OCD | 0.57(0.46 to 0.70) | 0 | 0.95(0.75 to 1.19) | 7 |  |  |
| Panic Disorder | 0.47(0.33 to 0.67) | 44 | 0.64(0.49 to 0.83) | 0 |  |  |
| Social Phobia | 0.66(0.51 to 0.86) | 25 | 0.81(0.65 to 1.03) | 0 |  |  |
| Specific Phobias^‡1^ | 0.86(0.72 to 1.03) | 0 | 1.04(0.86 to 1.26) | 0 | 1.03(0.85 to 1.25) | 0 |
| History of Depression^‡3^ | 0.73(0.63 to 0.85) | 43 | 0.80(0.68 to 0.94) | 35 | 0.86(0.72 to 1.02) | 31 |
| History of antidepressants | 0.76(0.65 to 0.89) | 23 | 0.83(0.7 to 0.98) | 26 |  |  |
| Any past Treatment | 0.73(0.62 to 0.87) | 26 | 0.8(0.67 to 0.96) | 30 |  |  |
| Functional Impairment^‡3^ | 0.65(0.54 to 0.78) | 42 | 0.9(0.76 to 1.07) | 21 | 0.94(0.79 to 1.12) | 22 |
| Hazardous Alcohol misuse^‡2^ | 0.78(0.59 to 1.03) | 23 | 0.86(0.67 to 1.10) | 0 | 0.86(0.67 to 1.11) | 0 |
| ^ρ^ adjusted for treatment allocation, age, and gender only; *adjusted for baseline depression scale z-score, age, gender, and treatment allocation; ^‡^additionally adjusted for: ^‡1^employment status; ^‡2^ marital status; ^‡3^ employment status and marital status. | | | | | | |

**Supplementary Table 9.** Outcomes at 6-8 months post-baseline (“mean difference” in z-score of depressive symptoms and percentage difference (%) in depressive symptoms) per unit increase in baseline prognostic indicators.

|  | **Adjusted for Treatment, Age and Gender^ρ^** | | | | **Additionally adjusted for depressive symptom severity*** | | | | **Additionally adjusted for employment status and/or marital status ^‡^** | | | |
| --- | --- | --- | --- | --- | --- | --- | --- | --- | --- | --- | --- | --- |
| **Prognostic Indicator** | **Mean difference (95%CI)** | **I^2^** | **%(95%CI)** | **I^2^** | **Mean difference (95%CI)** | **I^2^** | **%(95%CI)** | **I^2^** | **Mean difference (95%CI)** | **I^2^** | **%(95%CI)** | **I^2^** |
| Depressive symptom severity | 0.38(0.29 to 0.48) | 85 | 33.4(23.0 to 44.7) | 75 | 0.38(0.29 to 0.48) | 85 | 33.4(23.0 to 44.7) | 75 |  |  |  |  |
| CIS-R Total Score | 0.04(0.03 to 0.04) | 0 | 13.6(11.1 to 16.2) | 0 | 0.02(0.01 to 0.03) | 60 | 6.0(2.3 to 9.8) | 38 |  |  |  |  |
| Depressive Subscales Total^‡1^ | 0.08(0.06 to 0.11) | 77 | 27.9(16.3 to 40.6) | 64 | 0.04(0.01 to 0.06) | 70 | 8.8(-3.8 to 23.0) | 73 | 0.03(0.00 to 0.05) | 62 | 7.7(-3.7 to 20.4) | 67 |
| Anxiety Subscales Total^‡2^ | 0.04(0.02 to 0.05) | 83 | 11.2(4.1 to 18.8) | 78 | 0.02(0.00 to 0.03) | 81 | 3.4(-2.6 to 9.7) | 66 | 0.01(0.00 to 0.03) | 78 | 3.6(-2.4 to 9.9) | 66 |
| Compulsions Score | 0.10(0.05 to 0.16) | 54 | 6.4(3.0 to 9.9) | 1 | 0.04(-0.03 to 0.11) | 72 | 0.8(-3.8 to 5.7) | 50 |  |  |  |  |
| Compulsions Duration^‡1^ | 0.05(0.03 to 0.08) | 0 | 3.4(1.4 to 5.5) | 0 | 0.02(0.00 to 0.05) | 4 | 1.2(-1.2 to 3.7) | 31 | 0.02(0.00 to 0.05) | 14 | 1.3(-1.0 to 3.6) | 25 |
| Concentration Score | 0.16(0.10 to 0.21) | 75 | 9.7(5.8 to 13.9) | 31 | 0.07(0.02 to 0.13) | 73 | 3.3(0.2 to 6.5) | 0 |  |  |  |  |
| Concentration Duration | 0.10(0.04 to 0.17) | 79 | 8.1(1.9 to 14.7) | 79 | 0.06(0.01 to 0.10) | 62 | 4.6(-0.0 to 9.5) | 65 |  |  |  |  |
| Depression Score^‡3^ | 0.17(0.11 to 0.22) | 60 | 13.3(7.3 to 19.7) | 49 | 0.04(-0.02 to 0.10) | 62 | 2.6(-4.8 to 10.6) | 68 | 0.02(-0.06 to 0.10) | 65 | 2.5(-4.4 to 9.8) | 63 |
| Depressive Thoughts Score^‡1^ | 0.20(0.14 to 0.25) | 56 | 15.1(10.9 to 19.4) | 16 | 0.08(0.04 to 0.13) | 38 | 4.2(-1.8 to 10.5) | 56 | 0.06(0.01 to 0.11) | 29 | 3.6(-1.8 to 9.4) | 48 |
| Depression Duration^‡1^ | 0.12(0.07 to 0.18) | 56 | 10.1(4.4 to 16.1) | 55 | 0.08(0.03 to 0.13) | 55 | 6.4(0.8 to 12.2) | 57 | 0.07(0.02 to 0.12) | 49 | 5.6(0.5 to 11.0) | 50 |
| Fatigue Score^‡1^ | 0.08(0.02 to 0.14) | 52 | 4.8(-0.9 to 10.9) | 30 | 0.05(-0.01 to 0.10) | 43 | 2.9(-2.3 to 8.4) | 23 | 0.03(-0.02 to 0.08) | 25 | 3.2(-1.8 to 8.4) | 17 |
| Fatigue Duration | 0.11(0.06 to 0.15) | 40 | 8.3(4.8 to 11.9) | 3 | 0.08(0.04 to 0.11) | 22 | 6.0(2.8 to 9.4) | 0 |  |  |  |  |
| Generalised Anxiety Score^‡1^ | 0.09(0.06 to 0.12) | 0 | 5.0(2.3 to 7.8) | 0 | 0.03(0.00 to 0.06) | 0 | 0.6(-1.9 to 3.2) | 0 | 0.03(0.00 to 0.05) | 0 | 0.8(-1.8 to 3.3) | 0 |
| Generalised Anxiety Duration | 0.05(0.00 to 0.10) | 50 | 3.7(-0.4 to 7.9) | 52 | 0.03(-0.01 to 0.07) | 45 | 2.1(-1.6 to 6.0) | 50 |  |  |  |  |
| Health Anxiety Score^‡1^ | 0.13(0.06 to 0.20) | 74 | 9.8(5.1 to 14.6) | 48 | 0.06(0.02 to 0.11) | 48 | 4.5(1.3 to 7.8) | 0 | 0.04(0.00 to 0.09) | 42 | 3.7(0.5 to 6.9) | 0 |
| Health Anxiety Duration^‡3^ | 0.07(0.03 to 0.11) | 63 | 5.7(2.8 to 8.9) | 46 | 0.04(0.02 to 0.06) | 0 | 3.4(1.5 to 5.5) | 0 | 0.03(0.01 to 0.05) | 0 | 2.8(0.8 to 4.8) | 0 |
| Irritability Score^‡3^ | 0.11(0.06 to 0.16) | 52 | 6.6(3.0 to 10.2) | 16 | 0.04(0.00 to 0.09) | 50 | 1.4(-1.7 to 4.5) | 0 | 0.03(0.00 to 0.07) | 10 | 1.9(-1.2 to 5.2) | 0 |
| Irritability Duration^‡2^ | 0.06(0.00 to 0.12) | 74 | 4.4(-0.3 to 9.3) | 67 | 0.02(-0.03 to 0.07) | 65 | 1.8(-2.4 to 6.2) | 62 | 0.03(-0.03 to 0.08) | 66 | 2.0(-2.4 to 6.5) | 63 |
| Obsessions Score | 0.03(-0.01 to 0.07) | 53 | 0.9(-3.4 to 5.3) | 66 | -0.01(-0.05 to 0.03) | 51 | -2.3(-5.6 to 1.2) | 48 |  |  |  |  |
| Obsessions Duration | 0.03(0.00 to 0.05) | 0 | 1.9(-0.1 to 3.8) | 0 | -0.01(-0.03 to 0.02) | 19 | -0.3(-2.2 to 1.5) | 0 |  |  |  |  |
| Panic Score^‡2^ | 0.13(0.05 to 0.21) | 81 | 8.3(1.6 to 15.4) | 72 | 0.06(0.00 to 0.13) | 73 | 3.2(-2.3 to 8.9) | 60 | 0.06(-0.02 to 0.14) | 78 | 3.1(-2.3 to 8.9) | 59 |
| Panic Duration^‡1^ | 0.10(0.06 to 0.15) | 60 | 7.5(4.7 to 10.4) | 32 | 0.06(0.01 to 0.10) | 58 | 4.2(1.6 to 6.8) | 21 | 0.05(0.01 to 0.09) | 52 | 3.8(1.4 to 6.1) | 11 |
| Phobias Score^‡1^ | 0.15(0.07 to 0.22) | 75 | 9.4(4.3 to 14.7) | 54 | 0.08(0.01 to 0.15) | 76 | 4.2(-0.1 to 8.7) | 40 | 0.05(0.00 to 0.10) | 35 | 3.7(-0.1 to 7.6) | 26 |
| Phobias Duration^‡3^ | 0.07(0.03 to 0.11) | 59 | 5.5(3.0 to 8.1) | 37 | 0.03(0.00 to 0.06) | 42 | 2.7(0.4 to 5.1) | 28 | 0.03(0.00 to 0.06) | 31 | 2.3(0.3 to 4.3) | 10 |
| Sleep Score^‡3^ | 0.12(0.09 to 0.15) | 46 | 9.3(5.9 to 12.8) | 60 | 0.05(0.02 to 0.08) | 30 | 3.5(0.3 to 6.8) | 61 | 0.04(0.00 to 0.08) | 35 | 2.7(-0.5 to 5.9) | 50 |
| Sleep Duration^‡1^ | 0.09(0.05 to 0.14) | 69 | 7.4(2.3 to 12.7) | 74 | 0.06(0.01 to 0.11) | 73 | 4.8(-0.2 to 10.0) | 76 | 0.05(0.01 to 0.09) | 66 | 4.2(-0.4 to 8.9) | 71 |
| Somatic Score^‡3^ | 0.11(0.07 to 0.14) | 32 | 8.00(5.3 to 10.8) | 0 | 0.07(0.04 to 0.11) | 28 | 5.4(2.8 to 8.1) | 0 | 0.06(0.03 to 0.09) | 0 | 5.0(2.4 to 7.6) | 0 |
| Somatic Duration^‡3^ | 0.09(0.06 to 0.11) | 2 | 7.6(5.4 to 9.8) | 0 | 0.06(0.04 to 0.09) | 0 | 5.7(3.6 to 7.9) | 0 | 0.05(0.03 to 0.08) | 0 | 5.1(3.0 to 7.3) | 0 |
| Worry Score^‡1^ | 0.07(0.03 to 0.11) | 37 | 5.2(2.1 to 8.4) | 1 | 0.01(-0.02 to 0.04) | 0 | -0.2(-3.1 to 2.9) | 0 | 0.01(-0.02 to 0.04) | 0 | 0.5(-2.5 to 3.5) | 0 |
| Worry Duration | 0.07(0.02 to 0.11) | 49 | 6.2(1.9 to 10.6) | 55 | 0.04(-0.01 to 0.09) | 59 | 4.4(-0.1 to 9.1) | 64 |  |  |  |  |
| Average Duration of Anxiety^‡3^ | 0.23(0.14 to 0.32) | 71 | 19.0(11.5 to 27.1) | 58 | 0.12(0.04 to 0.21) | 63 | 10.8(3.4 to 18.7) | 58 | 0.11(0.04 to 0.18) | 54 | 9.7(3.3 to 16.5) | 45 |
| Number of Comorbid CMDs^‡3^ | 0.20(0.07 to 0.33) | 86 | 14.7(3.3 to 27.3) | 85 | 0.08(-0.03 to 0.19) | 80 | 5.8(-4.3 to 16.9) | 82 | 0.07(-0.03 to 0.17) | 78 | 4.9(-4.4 to 15.1) | 79 |
| Agoraphobia^‡1^ | 0.32(0.12 to 0.51) | 28 | 26.2(11.5 to 42.8) | 0 | 0.15(-0.01 to 0.32) | 13 | 11.7(-0.9 to 25.9) | 0 | 0.09(-0.06 to 0.24) | 0 | 6.9(-5.2 to 20.5) | 0 |
| CFS^‡2^ | 0.27(0.15 to 0.38) | 28 | 21.5(10.0 to 34.1) | 26 | 0.08(-0.05 to 0.21) | 49 | 7.2(-4.9 to 20.8) | 48 | 0.09(-0.05 to 0.22) | 48 | 7.6(-4.3 to 21.0) | 46 |
| GAD | 0.21(0.10 to 0.33) | 0 | 13.2(3.4 to 23.9) | 0 | 0.03(-0.08 to 0.13) | 0 | 0.4(-8.0 to 9.7) | 0 |  |  |  |  |
| MADD | -0.32(-0.42 to -0.22) | 0 | -21.4(-28.2 to -14.0) | 15 | -0.14(-0.24 to -0.04) | 5 | -9.4(-17.3 to -0.8) | 43 |  |  |  |  |
| OCD | 0.38(0.10 to 0.66) | 72 | 29.4(6.6 to 57.1) | 62 | 0.06(-0.17 to 0.29) | 58 | 3.4(-15.2 to 26.0) | 61 |  |  |  |  |
| Panic Disorder | 0.39(0.01 to 0.77) | 73 | 33.8(8.5 to 65.0) | 45 | 0.24(0.02 to 0.47) | 33 | 17.2(1.9 to 34.8) | 0 |  |  |  |  |
| Social Phobia | 0.22(0.06 to 0.38) | 0 | 14.8(1.9 to 29.4) | 0 | 0.11(-0.04 to 0.26) | 0 | 7.1(-4.8 to 20.4) | 4 |  |  |  |  |
| Specific Phobias^‡1^ | 0.06(-0.08 to 0.21) | 0 | 8.7(-2.9 to 21.6) | 0 | 0.00(-0.14 to 0.14) | 0 | 3.5(-7.3 to 15.5) | 0 | 0.03(-0.10 to 0.17) | 0 | 6.4(-4.6 to 18.9) | 0 |
| History of Depression^‡3^ | 0.19(0.10 to 0.28) | 43 | 9.3(-0.6 to 20.1) | 62 | 0.11(0.03 to 0.20) | 8 | 2.9(-6.2 to 12.8) | 47 | 0.08(-0.02 to 0.18) | 12 | 2.8(-6.2 to 12.7) | 43 |
| History of antidepressants | 0.19(0.11 to 0.28) | 0 | 12.0(-2.6 to 28.7) | 54 | 0.11(0.03 to 0.19) | 0 | 4.1(-9.4 to 19.6) | 56 |  |  |  |  |
| Any past Treatment | 0.20(0.10 to 0.30) | 10 | 11.7(-2.7 to 28.3) | 53 | 0.10(0.01 to 0.19) | 1 | 3.5(-9.5 to 18.4) | 52 |  |  |  |  |
| Functional Impairment^‡3^ | 0.30(0.15 to 0.45) | 61 | 24.5(13.5 to 36.6) | 24 | 0.09(-0.02 to 0.19) | 22 | 7.2(-1.2 to 16.4) | 0 | 0.08(-0.02 to 0.17) | 6 | 6.3(-2.1 to 15.4) | 0 |
| Hazardous Alcohol misuse^‡2^ | 0.03(-0.19 to 0.25) | 49 | -1.8(-15.1 to 13.6) | 23 | 0.00(-0.19 to 0.19) | 42 | -2.4(-14.4 to 11.2) | 15 | 0.00(-0.19 to 0.19) | 42 | -2.1(-15.0 to 11.8) | 21 |
| ^ρ^ adjusted for treatment allocation, age, and gender only; *adjusted for baseline depression scale z-score, age, gender, and treatment allocation; ^‡^additionally adjusted for: ^‡1^employment status; ^‡2^ marital status; ^‡3^ employment status and marital status | | | | | | | | | | | | |

**Supplementary Table 10.** Impact on amount of variance explained in depressive symptom scale scores at 3-4 post-baseline, modelled with the z-score and natural logarithm outcomes, when adding each variable in turn.

| **Models, adding each variable one at a time** | **Cumulative impact adding each variable one at a time** | |
| --- | --- | --- |
|  | z-score of depressive symptom scale scores adjusted R^2^ | log of depressive symptom scale scores adjusted R^2^ |
| Depressive symptom severity and covariates | 0.16 | 0.10 |
| Average anxiety duration | 0.22 | 0.14 |
| Depression Duration | 0.25 | 0.19 |
| Panic Disorder | 0.27 | 0.20 |
| History of antidepressants | 0.27 | 0.21 |
| **Final model** | 0.27 | 0.21 |
| Final model adjusted for depressive symptom severity, depression duration, average anxiety duration, panic disorder, history of antidepressants, treatment allocation, age, gender, employment status, and marital status. All models excluded data from AHEAD & HEALTHLINES | | |

**Supplementary Table 11.** Results of original analyses and corresponding sensitivity analyses.

| **Analysis** | **Change for Sensitivity Analysis** | **Pooled Effect Estimate** |
| --- | --- | --- |
| **z-score 3-4 months outcome** |  | **mean difference (95%CI)** |
| Depressive symptom severity**^ρ^** | Original Analysis using z-score of depressive symptoms at 3-4 months | 0.44(0.41 to 0.47) |
|  | bivariate meta-analysis using both 3-4 month and 6-8 month | 0.42(0.36 to 0.48) |
| Depressive symptom severity**^ρ^** | Analysis using all 11 studies irrespective of systematically missing data | 0.44(0.41 to 0.47) |
|  | Removing two studies with systematically missing data on many ‘disorder severity factors’ (AHEAD & HEALTHLINES) | 0.45(0.42 to 0.49) |
| Depressive symptom severity**^ρ^** | Original Analysis using z-score of depressive symptoms at 3-4 months in imputed data | 0.44(0.41 to 0.47) |
|  | Analysis using observed ‘un-imputed’ data | 0.43(0.39 to 0.47) |
| Anxiety Subscales Total^‡2^ | Original Analysis using z-score of depressive symptoms at 3-4 months | 0.04(0.03 to 0.05) |
|  | Analysis removing study contributing most to heterogeneity (TREAD) | 0.04(0.04 to 0.05) |
| Panic Score**^ρ^** | Original Analysis with all studies | 0.13(0.07 to 0.19) |
|  | Analysis removing study contributing most to heterogeneity (TREAD) | 0.15(0.1 to 0.2) |
| Phobias Score**^ρ^** | Original Analysis with all studies | 0.15(0.1 to 0.2) |
|  | Analysis removing study contributing most to heterogeneity (TREAD) | 0.17(0.14 to 0.2) |
| Number of Comorbid CMDs**^ρ^** | Original Analysis with all studies | 0.21(0.12 to 0.29) |
|  | Analysis removing study contributing most to heterogeneity (TREAD) | 0.24(0.19 to 0.3) |
| **log outcome at 3-4 months** |  | **%(95%CI)** |
| Depressive symptom severity**^ρ^** | Original Analysis using z-score of depressive symptoms at 3-4 months | 31(25 to 37) |
|  | Analysis removing two studies contributing most to heterogeneity (RESPOND and PANDA) | 31(25 to 36) |
| **z-score 6-8 months outcome** |  | **mean difference (95%CI)** |
| Depressive symptom severity**^ρ^** | Original Analysis with all studies | 0.38(0.29 to 0.48) |
|  | Analysis removing study contributing most to heterogeneity (IPCRESS) | 0.35(0.26 to 0.44) |
| Depressive Subscales Total^ρ^ | Original Analysis with all studies | 0.04(0.01 to 0.06) |
|  | Analysis removing study contributing most to heterogeneity (IPCRESS) | 0.05(0.03 to 0.07) |
| Anxiety Subscales Total* | Original Analysis with all studies | 0.02(0 to 0.03) |
|  | Analysis removing study contributing most to heterogeneity (TREAD) | 0.02(0.01 to 0.04) |
| Concentration Score | Original Analysis with all studies | 0.16(0.1 to 0.21) |
|  | Analysis removing study contributing most to heterogeneity (TREAD) | 0.18(0.13 to 0.22) |
| Concentration Duration | Original Analysis with all studies | 0.1(0.04 to 0.17) |
|  | Analysis removing study contributing most to heterogeneity (TREAD) | 0.12(0.05 to 0.19) |
| Panic Score**^‡^** | Original Analysis with all studies | 0.06(-0.02 to 0.14) |
|  | Analysis removing study contributing most to heterogeneity (TREAD) | 0.09(0.01 to 0.17) |
| Phobias Score* | Original Analysis with all studies | 0.05(0 to 0.1) |
|  | Analysis removing study contributing most to heterogeneity (TREAD) | 0.07(0.03 to 0.11) |
| Number of Comorbid CMDs**‡** | Original Analysis with all studies | 0.07(-0.03 to 0.17) |
|  | Analysis removing study contributing most to heterogeneity (TREAD) | 0.1(0 to 0.2) |
| **log outcome at 6-8 months** |  | **%(95%CI)** |
| Depressive symptom severity**^ρ^** | Original Analysis with all studies | 33.4(23.0 to 44.7) |
|  | Analysis removing study contributing most to heterogeneity (IPCRESS) | 30.0(20.3 to 40.5) |
| Anxiety Subscales Total**^ρ^** | Original Analysis with all studies | 2.3(0.9 to 3.6) |
|  | Analysis removing study contributing most to heterogeneity (TREAD) | 2.9(2.3 to 3.6) |
| Sleep Duration* | Original Analysis with all studies | 6.2(1.6 to 11.1) |
|  | Analysis removing study contributing most to heterogeneity (TREAD) | 7.6(2.8 to 12.7) |
| Number of Comorbid CMDs**‡** | Original Analysis with all studies | 12.9(2.5 to 24.3) |
|  | Analysis removing study contributing most to heterogeneity (TREAD) | 17.9(10.3 to 26.0) |
| **Remission outcome at 3-4 months** |  | **OR(95%CI)** |
| Number of Comorbid CMDs**^ρ^** | Original Analysis with all studies | 0.72(0.61 to 0.83) |
|  | Analysis removing study contributing most to heterogeneity (TREAD) | 0.67(0.6 to 0.75) |
| **Final Model Variables** |  |  |
| Depressive symptom severity**^‡^** | Original Analysis using z-score of depressive symptoms at 3-4 months | 29.94(22.74 to 37.58) |
|  | Analysis removing study contributing most to heterogeneity (RESPOND) | 32.07(24.82 to 39.74) |
| Depressive symptom severity⸷ | Original Analysis using log of depressive symptoms at 3-4 months | 25.24(19.01 to 31.79) |
|  | Analysis removing study contributing most to heterogeneity (RESPOND) | 27.11(21.18 to 33.33) |
| Average Anxiety Duration‡ | Original Analysis with all studies | 0.23(0.18 to 0.28) |
|  | Analysis removing study contributing most to heterogeneity (TREAD) | 0.25(0.21 to 0.29) |
| Depressive symptom severity**^‡^** | Original Analysis using log of depressive symptoms at 3-4 months in all 11 studies | 29.94(22.74 to 37.58) |
|  | Analysis removing study contributing most to heterogeneity (RESPOND) | 32.07(24.82 to 39.74) |
| Depressive symptom severity⸷ | Original Analysis using log of depressive symptoms at 3-4 months in all 11 studies | 25.24(19.01 to 31.79) |
|  | Analysis removing study contributing most to heterogeneity (RESPOND) | 27.11(21.18 to 33.33) |
| Z-score of main anxiety scale‡ | Original Analysis using z-score of depressive symptoms at 3-4 months in all 11 studies | 0.08(0.02 to 0.14) |
|  | Analysis removing study contributing most to heterogeneity (PANDA) | 0.07(0.04 to 0.7) |
| Z-score of main anxiety scale⸷ | Original Analysis using z-score of depressive symptoms at 3-4 months in all 11 studies | 0.08(0.02 to 0.14) |
|  | Analysis removing study contributing most to heterogeneity (PANDA) | 0.07(0 to 0.14) |
| Z-score of main anxiety scale‡ | Original Analysis using log of depressive symptoms at 3-4 months in all 11 studies | 5.5(1.62 to 9.53) |
|  | Analysis removing study contributing most to heterogeneity (PANDA) | 4.9(0.65 to 9.33) |
| Z-score of main anxiety scale⸷ | Original Analysis using log of depressive symptoms at 3-4 months in all 11 studies | 5.53(1.67 to 9.54) |
|  | Analysis removing study contributing most to heterogeneity (PANDA) | 4.91(0.63 to 9.37) |
| ^ρ^ adjusted for treatment allocation, age, and gender only; *additionally adjusted for depressive symptom severity; ‡additionally adjusted for covariates (employment status and/or marital status); ⸷additionally adjusting for disorder severity factors | | |

**Supplementary Table 12.** Association of prognostic indicators with outcomes adjusted for ‘disorder characteristics’, and impact in accuracy of models after adding each variable in turn. All variables in all studies.

| **Models, adding each variable one at a time** | **Cumulative impact adding each variable one at a time** | |
| --- | --- | --- |
|  | z-score of depressive symptom scale scores adjusted R^2^ | log of depressive symptom scale scores adjusted R^2^ |
| Depressive symptom severity and covariates | 0.16 | 0.10 |
| Z-score of main anxiety scale | 0.23 | 0.17 |
| History of antidepressants | 0.24 | 0.18 |
| **Final model†** | 0.24 | 0.18 |
| †Depressive symptom severity, z-score of baseline anxiety scale scores, history of depression, adjusted for treatment allocation, age, and gender | | |

| **Models, adding each variable one at a time** | **High on Factor/Present N(%)** | **Independent of treatment and depressive symptom severity‡** | | | | **Additionally adjusted for ‘disorder characteristics’†** | | | | |
| --- | --- | --- | --- | --- | --- | --- | --- | --- | --- | --- |
|  |  | **Mean difference (95%CI)*** | **I^2^** | **% difference^** | **I^2^** | | **Mean difference (95%CI)*** | **I^2^** | **% difference^** | **I^2^** |
| Depressive symptom severity and covariates | 2759(52.8) | 0.44(0.41 to 0.47) | 16 | 30.7(24.9 to 36.8) | 78 | | 0.37(0.33 to 0.42) | 38 | 26.0(19.7 to 32.7) | 76 |
| Z-score of main anxiety scale | 2575(49.3) | 0.12(0.09 to 0.15) | 72 | 7.3(3.8 to 10.9) | 52 | | 0.11(0.05 to 0.17) | 73 | 7.4(3.8 to 11.0) | 53 |
| History of antidepressants | 3436(65.8) | 0.10(0.04 to 0.16) | 24 | 3.7(-2.1 to 9.9) | 40 | | 0.08(0.01 to 0.15) | 27 | 2.9(-2.8 to 9.1) | 40 |
| *using z-score at 3-4 months as the outcome; ^ using the natural log of the depressive symptom scale scores at 3-4 months; **‡** adjusted for depressive symptom severity, treatment allocation, age, gender, employment status, and marital status. **†** adjusted for depressive symptom severity, z-score of baseline anxiety scale scores, history of antidepressants age, gender, and treatment allocation | | | | | | | | | | |
|  |  |  |  |  |  |  |  |  |  |  |

**Supplementary Table 13.** Impact in accuracy of models after adding each ‘disorder characteristics’ in turn. All variables in all studies.

**Supplementary Figure 1**. Forest plots of associations between baseline severity factors and the z-score of depressive symptom scales at 3-4 months post-baseline independent of treatment, depressive symptom severity, depressive ‘disorder characteristics’ present in the included studies, and covariates.

**Supplementary Figure 2**. Forest plots of associations between baseline severity factors and the z-score of depressive symptom scales at 3-4 months post-baseline independent of treatment, depressive symptom severity, depressive ‘disorder characteristics’ present in all studies, and covariates.

## Additional References

Beck, A. T., Steer, R. A., & Brown, G. K. (1996). Manual for the Beck Depression Inventory-II. *Manual for the Beck Depression Inventory-II*. San Antonio, Tx: Psychological Corporation.

Bower, P., Kontopantelis, E., Sutton, A., Kendrick, T., Richards, D. A., Gilbody, S., … Liu, E. T.-H. (2013). Influence of initial severity of depression on effectiveness of low intensity interventions: meta-analysis of individual patient data. *BMJ*, *346*(feb26 2), f540–f540. https://doi.org/10.1136/bmj.f540

Carter, G. C., Cantrell, R. A., Zarotsky, V., Haynes, V. S., Phillips, G., Alatorre, C. I., … Marangell, L. B. (2012). Comprehensive review of factors implicated in the heterogeneity of response in depression. *Depression and Anxiety*, *29*(4), 340–354. https://doi.org/10.1002/da.21918

Chekroud, A. M., Zotti, R. J., Shehzad, Z., Gueorguieva, R., Johnson, M. K., Trivedi, M. H., … Corlett, P. R. (2016). Cross-trial prediction of treatment outcome in depression: a machine learning approach. *The Lancet Psychiatry*, *3*(3), 243–250. https://doi.org/10.1016/S2215-0366(15)00471-X

Cox, B. D., Blaxter, M., Buckle, A. L. J., Fenner, N. P., Golding, J. F., Gore, M., … Wadsworth, M. E. J. Wichelow, M. (1987). *The Health and Lifestyle Survey*. Health Promotion Research Trust.

Cox, J. L., Holden, J. M., & Sagovsky, R. (1987). Detection of postnatal depression: development of the 10-item Edinburgh Postnatal Depression Scale. *The British Journal of Psychiatry*, *150*(6), 782–786.

Cristea, I. A., Karyotaki, E., Hollon, S. D., Cuijpers, P., & Gentili, C. (2019). Biological markers evaluated in randomized trials of psychological treatments for depression: a systematic review and meta-analysis. *Neuroscience & Biobehavioral Reviews*, *101*(101), 32–44. https://doi.org/10.1016/j.neubiorev.2019.03.022

Cuijpers, P., Karyotaki, E., Reijnders, M., & Huibers, M. J. H. (2018). Who benefits from psychotherapies for adult depression? A meta-analytic update of the evidence. *Cognitive Behaviour Therapy*, *47*(2), 91–106. https://doi.org/10.1080/16506073.2017.1420098

Dodd, S., Berk, M., Kelin, K., Mancini, M., & Schacht, A. (2013). Treatment response for acute depression is not associated with number of previous episodes: Lack of evidence for a clinical staging model for major depressive disorder. *Journal of Affective Disorders*, *150*(2), 344–349. https://doi.org/10.1016/j.jad.2013.04.016

Dodd, S., Berk, M., Kelin, K., Zhang, Q., Eriksson, E., Deberdt, W., & Craig Nelson, J. (2014). Application of the Gradient Boosted method in randomised clinical trials: Participant variables that contribute to depression treatment efficacy of duloxetine, SSRIs or placebo. *Journal of Affective Disorders*, *168*, 284–293. https://doi.org/10.1016/j.jad.2014.05.014

Driessen, E., Cuijpers, P., Hollon, S. D., & Dekker, J. J. M. (2010). Does Pretreatment Severity Moderate the Efficacy of Psychological Treatment of Adult Outpatient Depression ? A Meta-Analysis. *Journal of Consulting and Clinical Psychology*, *78*(5), 668–680. https://doi.org/10.1037/a0020570

Ebrahim, S., Montoya, L., Truong, W., Hsu, S., Kamal el Din, M., Carrasco-Labra, A., … Guyatt, G. H. (2012). Effectiveness of Cognitive Behavioral Therapy for Depression in Patients Receiving Disability Benefits: A Systematic Review and Individual Patient Data Meta-Analysis. *PLoS ONE*, *7*(11). https://doi.org/10.1371/journal.pone.0050202

Fisher, D. J. (2015). Two-stage individual participant data meta-analysis and generalized forest plots. *The Stata Journal*, *2*, 369–396.

Fournier, J. C., DeRubeis, R. J., Hollon, S. D., Dimidjian, S., Amsterdam, J. D., Shelton, R. C., & Fawcett, J. (2010). Antidepressant Drug Effects and Depression Severity. *JAMA*, *303*(1), 47. https://doi.org/10.1001/jama.2009.1943

Gariepy, G., Honkaniemi, H., & Quesnel-Vallee, A. (2016). Social support and protection from depression : systematic review of current findings in Western countries. *British Journal of Psychiatry*, *209*, 284–293. https://doi.org/10.1192/bjp.bp.115.169094

Goldberg, D. (1992). *General Health Questionnaire (GHQ-12) NFER*. Nelson Publishing Co. Ltd, Windsor, UK.

Guyatt, G. H., Oxman, A., Vist, G., Kunz, R., Falck-Ytter, Y., Alonso-Coello, P., & Schünemann, H. J. (2008). GRADE an emerging consensus on rating quality of evidence and strength of recommendations. *British Medical Journal*, *336*(7650), 924–926. https://doi.org/10.1136/bmj.39489.470347.AD

Haq, A., Sitzmann, A., Goldman, M., Maixner, D., & Mickey, B. (2015). Response of depression to electroconvulsive therapy: a meta-analysis of clinical predictors. *Journal of Clinical Psychiatry*, *76*(10), 1374–1384.

Hayden, J. A., Windt, D. A. Van Der, Cartwright, J. L., Côté, P., & Bombardier, C. (2013). Assessing Bias in Studies of Prognostic Factors. *Annals of Internal Medicine*, *158*, 280–286.

Herdman, M., Gudex, C., Lloyd, A., Janssen, M. F., Kind, P., Parkin, D., … Badia, X. (2011). Development and preliminary testing of the new five-level version of EQ-5D ( EQ-5D-5L ). *Quality of Life Research*, 1727–1736. https://doi.org/10.1007/s11136-011-9903-x

Higgins, J. P. T., & Green, S. (2011). *Cochrane Handbook for Systematic Reviews of Interventions* (J. P. T. Higgins & S. Geen, Eds.). Chichester: The Cochrane Collaboration.

Holmes, T. H., & Rahe, R. H. (1967). The social readjustment rating scale. *Journal of Psychosomatic Research*, *11*(2), 213–218.

Horton, N. J., & Lipsitz, S. R. (2001). Multiple Imputation in Practice: Comparison of Software Packages for Regression Models With Missing Variables. *The American Statistician*, *55*(3), 244–254. https://doi.org/10.1198/000313001317098266

Hout, B. Van, Janssen, M. F., Feng, Y., Kohlmann, T., & Busschbach, J. (2012). Interim Scoring for the EQ-5D-5L : Mapping the EQ-5D-5L to EQ-5D-3L. *JVAL*, *15*(5), 708–715. https://doi.org/10.1016/j.jval.2012.02.008

Iovieno, N., Tedeschini, E., Ameral, V. E., Rigatelli, M., & Papakostas, G. I. (2011). Antidepressants for major depressive disorder in patients with a co-morbid axis-III disorder: A meta-analysis of patient characteristics and placebo response rates in randomized controlled trials. *International Clinical Psychopharmacology*, *26*(2), 69–74. https://doi.org/10.1097/YIC.0b013e328340775e

Johnsen, T. J., & Friborg, O. (2015). The effects of cognitive behavioral therapy as an anti-depressive treatment is falling: A meta-analysis. *Psychological Bulletin*, *141*(4), 747–768. https://doi.org/10.1037/bul0000015

Kampman, O., & Poutanen, O. (2011). Can onset and recovery in depression be predicted by temperament? A systematic review and meta-analysis. *Journal of Affective Disorders*, *135*(1–3), 20–27. https://doi.org/10.1016/j.jad.2010.12.021

Karyotaki, E., Riper, H., Twisk, J., Hoogendoorn, A., Kleiboer, A., Mira, A., … Cuijpers, P. (2017). Efficacy of Self-guided Internet-Based Cognitive Behavioral Therapy in the Treatment of Depressive Symptoms. *JAMA Psychiatry*, *74*(4), 351. https://doi.org/10.1001/jamapsychiatry.2017.0044

Kendrick, T., Simons, L., Gray, A., Lathlean, J., Pickering, R., Harris, S., … Thompson, C. (2005). A trial of problem-solving by community mental health nurses for anxiety, depression and life difficulties among general practice patients. The CPN-GP study. *Health Technology Assessment*, *9*(37).

Kroenke, K., Spitzer, R. L., & Williams, J. B. W. (2001). The PHQ-9. *Journal of General Internal Medicine*, *16*(9), 606–613. https://doi.org/10.1046/j.1525-1497.2001.016009606.x

Lewis, G., Pelosi, A. J., Araya, R., & Dunn, G. (1992). Measuring psychiatric disorder in the community : a standardized assessment for use by lay interviewers. *Psychological Medicine*, *22*, 465–486.

Lin, X.-Q., Wang, P., Cai, W.-K., Xu, G.-L., Yang, M., Zhou, M.-D., … He, G.-H. (2019). The Associations Between CYP2D6 Metabolizer Status and Pharmacokinetics and Clinical Outcomes of Venlafaxine: A Systematic Review and Meta-Analysis. *Pharmacopsychiatry*, *52*(05), 222–231.

Marwood, L., Wise, T., Perkins, A. M., & Cleare, A. J. (2018). Meta-analyses of the neural mechanisms and predictors of response to psychotherapy in depression and anxiety. *Neuroscience and Biobehavioral Reviews*, *95*(September), 61–72. https://doi.org/10.1016/j.neubiorev.2018.09.022

Morris, B. H., Bylsma, L. M., & Rottenberg, J. (2009). Does emotion predict the course of major depressive disorder? A review of prospective studies. *British Journal of Clinical Psychology*, *48*(3), 255–273. https://doi.org/10.1348/014466508X396549

Morris, T. P., White, I. R., & Royston, P. (2014). Tuning multiple imputation by predictive mean matching and local residual draws. *BMC Medical Research Methodology*, *14*(1), 75. https://doi.org/10.1186/1471-2288-14-75

Nakabayashi, T., Hara, A., & Minami, H. (2018). Impact of demographic factors on the antidepressant effect: A patient-level data analysis from depression trials submitted to the Pharmaceuticals and Medical Devices Agency in Japan. *Journal of Psychiatric Research*, *98*, 116–123. https://doi.org/10.1016/j.jpsychires.2017.12.019

Noma, H., Furukawa, T. A., Maruo, K., Imai, H., Shinohara, K., Tanaka, S., … Cipriani, A. (2019). Exploratory analyses of effect modifiers in the antidepressant treatment of major depression: Individual-participant data meta-analysis of 2803 participants in seven placebo-controlled randomized trials. *Journal of Affective Disorders*, *250*(December 2018), 419–424. https://doi.org/10.1016/j.jad.2019.03.031

Paykel, E. S. (1994). Life events, social support and depression. *Acta Psychiatrica Scandinavica*, *89*(377, Suppl), 50–58. Retrieved from http://ovidsp.ovid.com/ovidweb.cgi?T=JS&PAGE=reference&D=psyc3&NEWS=N&AN=1994-37529-001

Piccinelli, M., Tessari, E., Bortolomasi, M., Piasere, O., Semenzin, M., Garzotto, N., & Tansella, M. (1997). Efficacy of the AUDIT as a screening tool for hazardous alcohol intake and related disorders in primary care: a validity study. *British Medical Journal*, *314*, 314–320.

Proudfoot, J., Goldberg, D., Mann, A., Everitt, B., Marks, I. M., & Gray, J. A. G. R. (2003). Computerized, interactive, multimedia cognitive-behavioural program for anxiety and depression in general practice. *Psychological Medicine*, 217–227.

Resche-Rigon, M., White, I. R., Bartlett, J. W., Peters, S. A. E., & Thompson, S. G. (2013). Multiple imputation for handling systematically missing confounders in meta-analysis of individual participant data. *Statistics in Medicine*, *32*(28), 4890–4905. https://doi.org/10.1002/sim.5894

Royston, P. (2009). Multiple imputation of missing values: Further update of ice, with an emphasis on categorical variables. *The Stata Journal*, *9*(3), 466–477.

Royston, P., & White, I. R. (2011). Multiple Imputation by Chained Equations (MICE): Implementation in Stata. *Journal of Statistical Software*, *45*(4), 1–20. https://doi.org/10.1002/mpr.329.Multiple

Schoemaker, J. H., Kilian, S., Emsley, R., & Vingerhoets, A. J. J. M. (2018). Factors associated with placebo response in depression trials: A systematic review of published meta-analyses (1990–2017). *Neurology Psychiatry and Brain Research*, *30*(April), 12–21. https://doi.org/10.1016/j.npbr.2018.04.002

Sockol, L. E. (2018). A systematic review and meta-analysis of interpersonal psychotherapy for perinatal women. *Journal of Affective Disorders*, *232*(August 2017), 316–328. https://doi.org/10.1016/j.jad.2018.01.018

Spitzer, R. L., Kroenke, K., Williams, J. B. W., & Löwe, B. (2006). A Brief Measure for Assessing Generalized Anxiety Disorder. *Archives of Internal Medicine*, *166*(10), 1092. https://doi.org/10.1001/archinte.166.10.1092

StataCorp LP. (2017). *Stata Base Reference Manual - Release 15*. College Station, Texas: Stata Press.

Stefan Kloiber, Ripke, S., Kohli, M. A., Reppermund, S., Salyakina, D., Uher, R., … Lucae, and S. (2013). Resistance to antidepressant treatment is associated with polymorphisms in the leptin gene, decreased leptin mRNA expression, and decreased leptin serum levels. *European Neuropsychopharmacology*, *23*(7), 653–662. https://doi.org/10.1161/CIRCULATIONAHA.110.956839

Steinert, C., Hofmann, M., Kruse, J., & Leichsenring, F. (2014). The prospective long-term course of adult depression in general practice and the community. A systematic literature review. *Journal of Affective Disorders*, *152*–*154*(1), 65–75. https://doi.org/10.1016/j.jad.2013.10.017

Sugarman, M. A., Loree, A. M., Baltes, B. B., Grekin, E. R., & Kirsch, I. (2014). The efficacy of paroxetine and placebo in treating anxiety and depression: A meta-analysis of change on the Hamilton Rating Scales. *PLoS ONE*, *9*(8). https://doi.org/10.1371/journal.pone.0106337

Wang, J., Mann, F., Lloyd-Evans, B., Ma, R., & Johnson, S. (2018). Associations between loneliness and perceived social support and outcomes of mental health problems : a systematic review. *BMC Psychiatry*, *18*(156), 1–16.

Weitz, E. S., Hollon, S. D., Twisk, J., Van Straten, A., Huibers, M. J. H., David, D., … Cuijpers, P. (2015). Baseline depression severity as moderator of depression outcomes between cognitive behavioral therapy vs pharmacotherapy: An individual patient data meta-analysis. *JAMA Psychiatry*, *72*(11), 1102–1109. https://doi.org/10.1001/jamapsychiatry.2015.1516

White, I. R. (2011). Multivariate Random-effects Meta-regression: Updates to Mvmeta. *The Stata Journal: Promoting Communications on Statistics and Stata*, *11*(2), 255–270. https://doi.org/10.1177/1536867X1101100206

Zigmond, A. S., & Snaith, R. P. (1983). The hospital anxiety and depression scale. *Acta Psychiatrica Scandinavica*, *67*(6), 361–370.
